# Microbial, proteomic, and metabolomic profiling of the estrous cycle in wild house mice

**DOI:** 10.1128/spectrum.02037-23

**Published:** 2024-01-03

**Authors:** Tereza Matějková, Alica Dodoková, Jakub Kreisinger, Pavel Stopka, Romana Stopková

**Affiliations:** 1 Department of Zoology, Faculty of Science, Charles University, BIOCEV, Vestec, Czechia; Chengdu University, Chengdu, Sichuan, China

**Keywords:** vaginal, oral, microbiome, saliva, estrus, estrous cycle, *Pasteurellaceae*, 16S RNA sequencing, proteome, *Mus musculus*

## Abstract

Symbiotic microbial communities affect the host immune system and produce molecules contributing to the odor of an individual. In many mammalian species, saliva and vaginal fluids are important sources of chemical signals that originate from bacterial metabolism and may act as honest signals of health and reproductive status. In this study, we aimed to define oral and vaginal microbiomes and their dynamics throughout the estrous cycle in wild house mice. In addition, we analyzed a subset of vaginal proteomes and metabolomes to detect potential interactions with microbiomes. 16S rRNA sequencing revealed that both saliva and vagina are dominated by *Firmicutes* and *Proteobacteria* but differ at the genus level. The oral microbiome is more stable during the estrous cycle and most abundant bacteria belong to the genera *Gemella* and *Streptococcus*, while the vaginal microbiome shows higher bacterial diversity and dynamics during the reproductive cycle and is characterized by the dominance of *Muribacter* and *Rodentibacter*. These two genera cover around 50% of the bacterial community during estrus. Proteomic profiling of vaginal fluids revealed specific protein patterns associated with different estrous phases. Highly expressed proteins in estrus involve the keratinization process thus providing estrus markers (e.g., Hrnr) while some proteins are downregulated such as immune-related proteins that limit bacterial growth (Camp, Clu, Elane, Lyz2, and Ngp). The vaginal metabolome contains volatile compounds potentially involved in chemical communication, for example, ketones, aldehydes, and esters of carboxylic acids. Data integration of all three OMICs data sets revealed high correlations, thus providing evidence that microbiomes, host proteomes, and metabolomes may interact.

**IMPORTANCE:**

Our data revealed dynamic changes in vaginal, but not salivary, microbiome composition during the reproductive cycle of wild mice. With multiple OMICs platforms, we provide evidence that changes in microbiota in the vaginal environment are accompanied by changes in the proteomic and metabolomics profiles of the host. This study describes the natural microbiota of wild mice and may contribute to a better understanding of microbiome-host immune system interactions during the hormonal and cellular changes in the female reproductive tract. Moreover, analysis of volatiles in the vaginal fluid shows particular substances that can be involved in chemical communication and reproductive behavior.

## INTRODUCTION

Symbiotic bacteria are involved in many processes within the host body. They have a protective role in all mucosal tissues, activate the innate immune system, provide molecules important for biochemical pathways, and produce volatile substances that can serve as olfactory signals in chemical communication. Olfactory signals, produced by the host body and its microbiome, are important sources of information and they affect the behavior and physiology of the animal receiver. Microbiome research in recent years highlights the link between many of the modern multifactorial diseases and altered microbiome shifts called dysbiosis (1). Laboratory mice are widely used in microbiome research; however, recent studies have shown that behavioral, physiological, and immune system responses are different compared to their wild counterparts (2–5). The sanitized environment strongly influences natural microbiome composition, which is the main force of immune system evolution and thus laboratory mice living in a less diverse microbial world may not phenocopy natural immune responses (6). It is therefore essential to characterize natural commensal microbiota in immunologically important epithelial barrier sites such as the oral cavity or vagina to better understand their importance and their contribution to tissue homeostasis. In addition, the oral cavity and vaginal openings are the first sites of inspection in many mammals and are thus important in social communication.

In most mammals, the estrous cycle exhibits four consecutive phases—proestrus, estrus—a period during which ovulation occurs, metestrus—characteristic of leucocyte invasion and diestrus (7–10), and each of these phases is characterized by fluctuating hormonal levels (11, 12), representation of the specific cell types (8, 10), mucus consistency and also changing protein levels (13–15). The general composition of the vaginal microbiome has been studied in various mammalian species (16) including humans (11), primates (12, 17), and rodents (18, 19) and it seems to be species specific to some extent. There is clear evidence for increased bacterial abundance during the estrus phase due to the dominant occurrence of keratinized cells that are massively invaded by some bacterial species. This may be the force that could lead to the growth of some phase-specific bacterial species, potentially producing molecules that serve as “honest signals” which reliably convey reproductive receptivity to the receiver (20) of reproductive receptivity. However, the changes in bacterial composition during the estrous cycle are still understudied and various publications present inconsistent conclusions (12, 21, 22).

The oral microbiome is more extensively exposed to the surrounding environment in comparison to the vaginal microbiome. Oral microbiota and its bioactive molecules also help the host in the first line of defense against invading pathogens and can signal the health condition of an animal to its conspecifics during social interactions (23). In mice, the first contact usually starts with mouse sniffing and licking; therefore, it is highly likely that saliva may serve as a source of important chemical cues for olfactory communication (24).

Microbiome communities are regulated by mutual interactions of bacterial species as well as by the host defense system involving various antimicrobial peptides and proteins inhibiting bacterial growth (e.g., siderocalins) (25). Neutrophils and epithelial cells play central role in secreting natural antimicrobial peptides that protect the reproductive tract against bacterial invasions. The spectrum of components with antimicrobial functions was detected in the female lower reproductive tract including, for example, defensins, chemokines, cathelicidins, lysozymes, lactoferrins, or lipocalins, and there is evidence that their activity is regulated by the production of sex hormones (14, 26, 27), changes in pH, salt concentration, or presence of sperm (28). Those studies that focused on changes in all proteins during the estrous cycle demonstrated particular differentially abundant proteins that are dominant in ovulatory (namely members of heat shock protein family, Hsp) or luteal phase (e.g., lactoferrin Ltf, cathelicidin Camp, neutrophilic granule protein Ngp) (14, 29, 30). Some proteins that are elevated during estrus may be involved in gamete interaction and enhance the sperm viability in the oviduct around ovulation (31) and at the same time decreased levels of some antimicrobial proteins may contribute to mating success. The predominance of keratinized cells and the absence of neutrophils during estrus may be the key factors of the highest bacterial abundance at this stage, documented in various mammalian species (32). Postovulatory increase in progesterone causes neutrophil infiltration in the reproductive tract (33) and leads to a higher production of antimicrobial proteins that can regulate bacterial growth. Therefore, hormonally induced changes in cytology profile during the estrous cycle associated with changes in bacterial abundance may be related to the observed fluctuation of antimicrobial proteins.

Bacteria themselves produce volatile metabolites resulting from catabolic processes including, for example, fermentation or lipid degradation, and together with metabolites originating from host interaction may produce distinct “volatile metabolic fingerprints” (34). Such volatile organic compounds (VOCs) serve as olfactory cues providing information about the condition of an individual, species, health, nutrition, sex, age, or reproductive state (35–39) and may directly influence behavior and social interactions. In rodents, urine, saliva, and vaginal fluids are the main sources of VOCs enabling individual recognition and influencing the behavior of the receiver. These signals are detected mainly by the vomeronasal organ and project to the accessory olfactory bulb where they have stable representations within neural circuits (40, 41). Urine serves as landmarks with sustained release of VOCs (42, 43), while in direct contact with individuals, orofacial and anogenital areas are explored during the first encounter (24, 44). For males, assessing the receptive phase of the female reproductive cycle is an important ability that increases their fitness *via* reproductive success. In some species (e.g., primates), ovulation is signaled by visual changes in reproductive organs (45) but in many species, males mainly rely on chemical signals and behavioral cues.

Microbiome dynamics during the estrous cycle and the contribution of host-derived proteins and volatile metabolites to the characterization of distinct phases of the reproductive cycle were the main scope of this study. We compared the bacterial composition of the oral and vaginal samples collected over the whole estrous cycle of wild-caught mice (*Mus musculus musculus*). Subsequently, we assessed whether the community structure of oral and vaginal microbiota was correlated with the dynamics of the estrous cycle, and, using differential abundance analysis, we searched for bacteria that were specific for each phase of the cycle. Abrupt changes in host-specific mechanisms regulating microbial populations, in addition to systematic changes in the abundance of specific bacterial taxa, can also lead to a stochastic response characterized by individual-specific rather than population-specific changes in the abundance of bacterial taxa ultimately manifesting in higher community dispersion (i.e., interindividual variation) among hosts. The latter pattern has often been observed in diseased cohorts, in which the host was unable to properly manage its microbial symbionts (46, 47). Therefore, we addressed the question of how the estrous cycle affects the variation in oral and vaginal microbiota between hosts and whether this variation can be explained by physiological differences at the proteome level. Analysis of mouse proteome of vaginal samples provided differentially abundant proteins that follow microbiome changes during the estrous cycle and may play roles in the regulation of microbiota. Finally, we focused on volatile molecules that could be of bacterial origin and thus can serve as honest scent signals in advertising the female reproductive state. Our study deepens the knowledge of the natural vaginal microbiome of wild living mice and its dynamics during the estrous cycle and establishes a solid platform for further research in the relationship between host-microbiota and reproduction-related chemical communication.

## RESULTS

In this study, we used three datasets—oral and vaginal microbiomes, host vaginal proteome, and volatile vaginal metabolome described in detail in Materials and methods. The data structure reflects two types of secretions (oral vs vaginal) and also phases of the mouse estrous cycle.

### Differences between oral and vaginal microbiota

**Fig 1:**
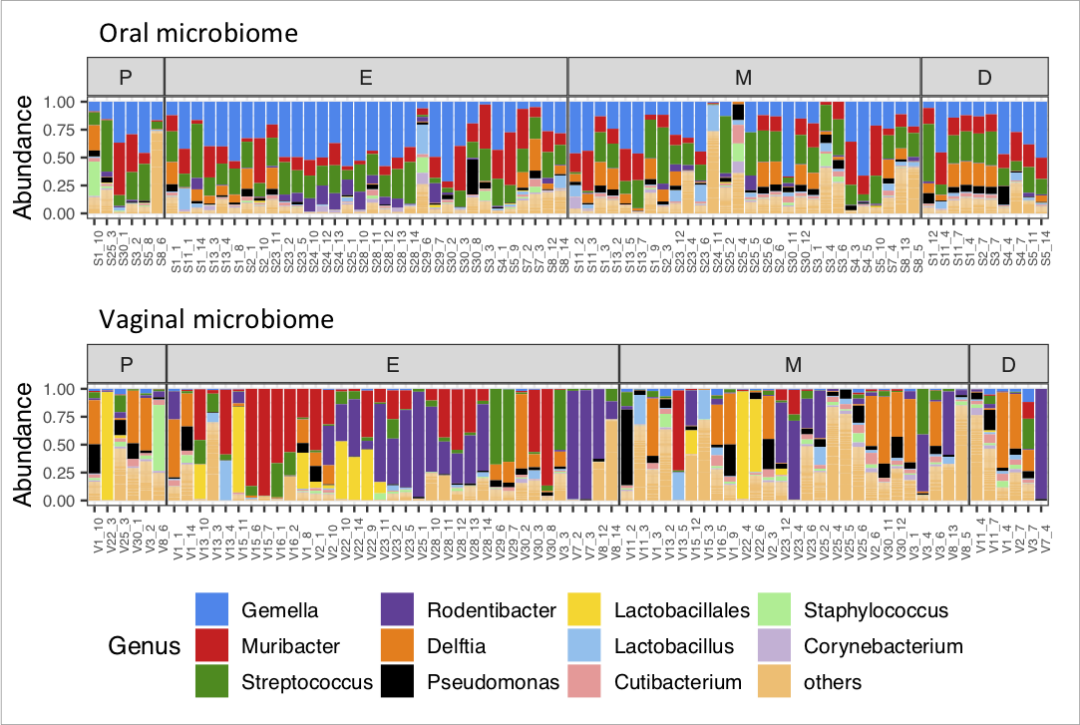
Relative representation of bacterial genera in oral and vaginal samples. Graphical depiction of the relative abundance of dominant bacterial genera in oral and vaginal samples during different phases of the estrous cycle (P, E, M, D = proestrus, estrus, metestrus, diestrus). *Firmicutes* (*Gemella, Streptococcus*) dominate the oral microbiome while *Proteobacteria* (*Rodentibacter, Muribacter, Delftia*) characterize the vaginal microbiome of female wild mice.

Microbiome analysis of oral and vaginal samples revealed fundamental differences in the composition of bacterial taxa in the two fluid types. Although *Firmicutes* and *Proteobacteria* were the most abundant phyla in both types of samples, the relative abundance of dominant bacterial genera was generally different in oral and vaginal samples (Fig. 1).

#### Alpha diversity

**Fig 2:**
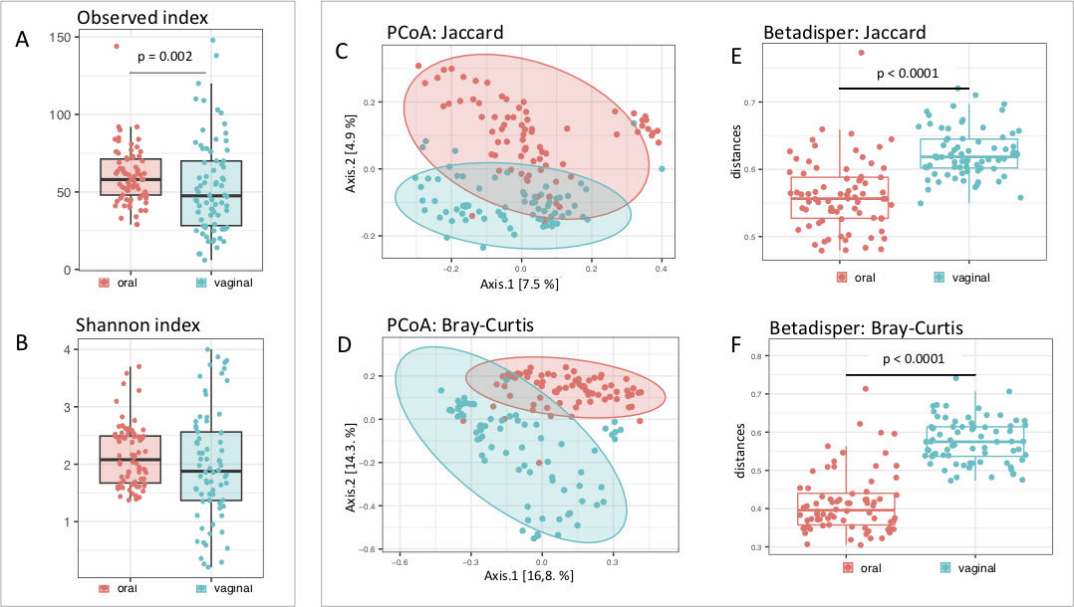
Alpha and beta diversity of oral and vaginal samples. The comparison of vaginal and oral samples on the level of alpha diversity shows a higher number of observed OTU in oral samples (**A**) but the Shannon index (**B**) is similar for both sample types. Principal Coordinate Analysis (PCoA) ordination revealed that the microbiota composition (i.e., beta diversity) varies between oral and vaginal samples according to both OTU absence/presence-based dissimilarities (Jaccard) and relative abundance-based dissimilarities (Bray-Curtis) (**C and D**). Moreover, vaginal microbiota exhibit higher heterogeneity in composition (**E and F**).

On the level of alpha diversity, vaginal microbiota exhibited a decreased number of observed operational taxonomic Units (OTUs) [linear mixed model (LMM): ΔD.F =1, χ^2^ = 9.6071, *P* = 0.001938] but not Shannon diversity (LMM: ΔD.F =1, χ^2^ = 1.3094, *P* = 0.2525) and higher variation compared to oral microbiota (Variance ratio F test: F_73,75_ = 4.1454, *P* < 0.0001 and F_73,75_ = 2.736, *P* < 0.0001 for Observed and Shannon diversity respectively) and imply higher heterogeneity in vaginal microbiome (Fig. 2A and B).

#### Beta diversity

Beta diversity in oral and vaginal samples showed systematic differences in composition [Multivariate Distance Matrix Regression (MDMR): D.F. = 1, test. stat = 7.00, *P* < 0.0001 and D.F. = 1, test. stat = 22.6, *P* < 0.0001 for Jaccard and Bray-Curtis dissimilarities, respectively). Principal Coordinate Analysis (PCoA) for both prevalence-based (Jaccard) and abundance-based (Bray-Curtis) dissimilarities showed that oral and vaginal samples formed separate clusters with minimal overlap between each other (Fig. 2C and D). Higher compositional homogeneity of oral microbiota in comparison to the vaginal microbiota is revealed by LMMs including distances of each sample to group-specific centroids as a response for both Jaccard (LMM: ΔD.F =1, χ^2^ = 86.873, *P* < 0.0001) and Bray-Curtis (LMM: ΔD.F =1, χ^2^ = 155.29, *P* < 0.0001) distances (Fig. 2E and F).

#### Representation of bacterial genera

**Fig 3:**
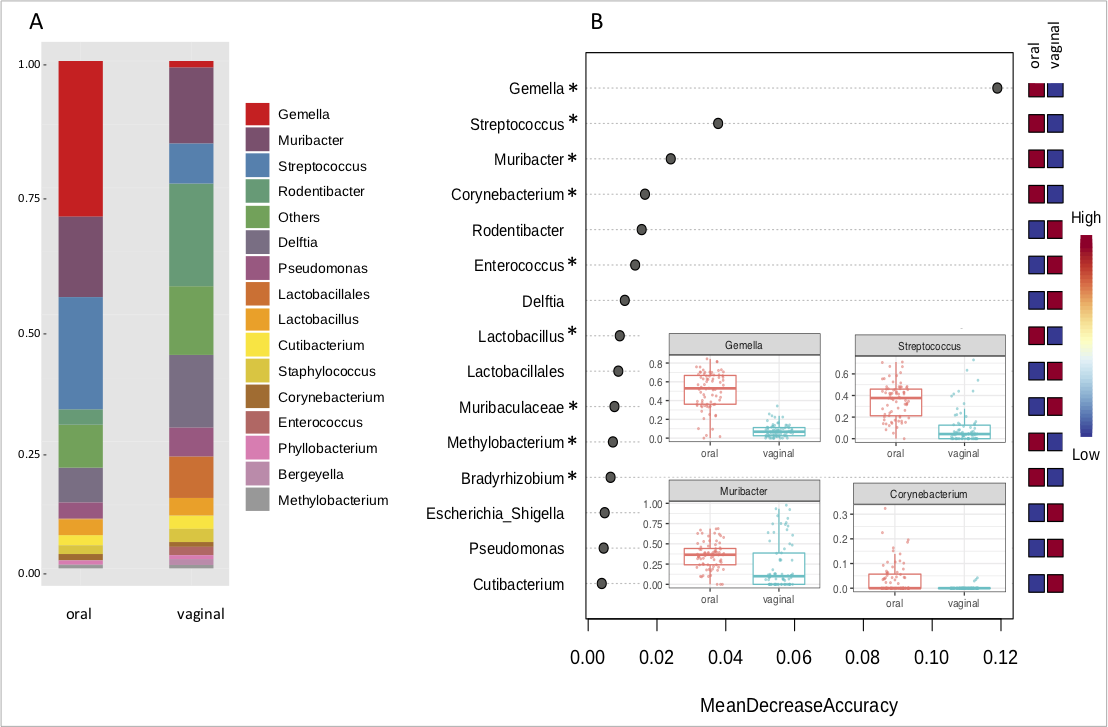
Significant differences in microbiota on the genus level between oral and vaginal samples. Relative average abundances of the most represented bacterial genera in oral and vaginal samples are graphically depicted in bar plot (**A**). Random Forest (**B**) demonstrates the most significant bacterial genera overrepresented in oral (*Gemella, Streptococcus, Muribacter, Corynebacterium, Lactobacillus, Methylobacterium, Bradyrhizobium*) and vaginal (*Rodentibacter, Enterococcus, Delftia, Lactobacillales*, *Muribaculaceae*) microbiome. Significant results of ANCOM analysis are marked with a star.

Based on the genus-level classification, the oral microbiome is characterized by the consistent high abundance of three major bacterial genera—*Gemella*, *Streptococcus,* and *Muribacter*. These taxons occurred at relatively high levels across all oral samples, forming usually more than 50% out of all sample reads, and were also overrepresented in oral microbiota in comparison to vaginal microbiota. The vaginal microbiome is predominantly formed by the presence of two members of the *Pasteurellaceae* family—*Rodentibacter* and *Muribacter,* accompanied by *Delftia* and *Streptococcus*. Contrary to the oral microbiome, the composition of the vaginal microbiome is more heterogeneous with a considerable contribution of unspecified bacteria from *Lactobacilalles* order and various minor bacterial species (labeled as Others) (Fig. 3A).

The most important bacterial taxa distinguishing both sample types are revealed by Random Forest analysis (Fig. 3B). In addition to the top three genera overrepresented in the oral microbiome (*Gemella*, *Streptococcus,* and *Muribacter*), ANCOM (Analysis of Composition of Microbiomes) identified other bacterial taxa exhibiting significantly increased abundance in oral compared to vaginal samples (*Corynebacterium*, *Lactobacillus*, *Methylobacterium,* and *Bradyrhizobium*). On the other hand, more heterogeneous vaginal microbiota is represented by *Rodentibacter, Delftia, Enterococcus*, *Lactobacillales,* and *Muribaculaceae*, however, probably due to high variation across all the samples, *Enterococcus* and unassigned *Muribaculaceae* are the only taxa significantly more represented in vaginal microbiomes (based on ANCOM analysis).

### Dynamics of oral and vaginal microbiota during the estrous cycle

#### Alpha diversity

**Fig 4:**
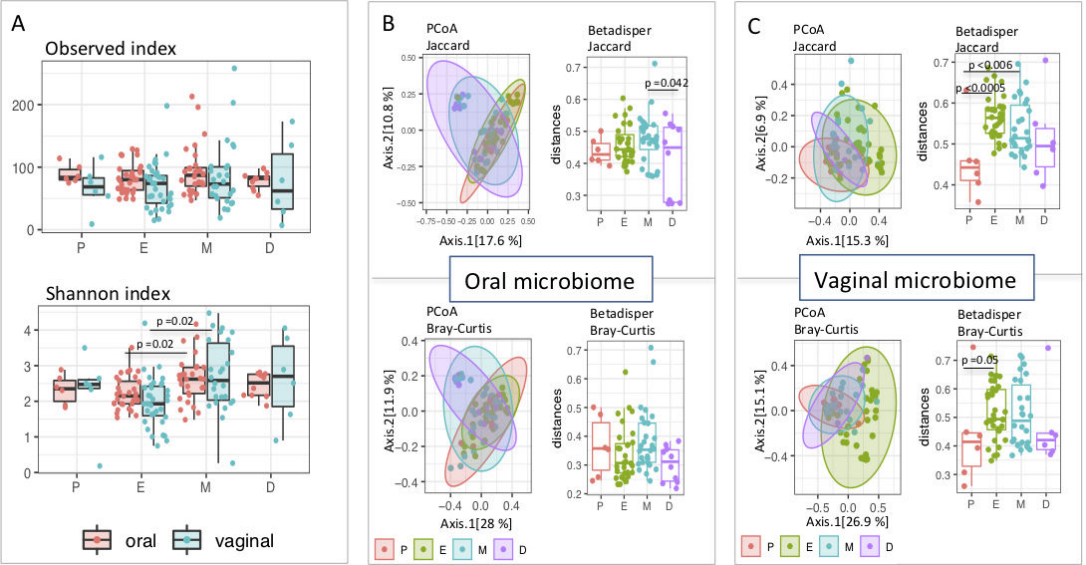
Alpha and beta diversity of oral and vaginal samples in individual phases of the estrous cycle. Alpha diversity during phases of the estrous cycle is stable on the level of observed OTU; however, the Shannon index is lowered in estrus compared to metestrus which indicates inequality between OTU abundances (**A**).PCoA showed greater changes in the composition of the vaginal microbiota across the estrous cycle than in the oral microbiota, and the vaginal microbiota also exhibited more pronounced changes in interindividual compositional variation (**B and C**). (P, E, M, D = phases of estrous cycle; proestrus, estrus, metestrus, diestrus).

Oral and vaginal samples did not differ in the number of observed OTUs in each phase of the cycle. However, linear mixed models revealed a significant effect of the phase of the estrus cycle for the Shannon index for both-oral and vaginal diversity (LMM oral: ΔD.F=3, χ^2^ = 10.285, *P* = 0.01629, LMM vaginal: ΔD.F=3, χ^2^ = 9.3765, *P* = 0.02468). Pairwise post-hoc Tukey test showed that Shannon diversity was significantly lower during estrus in comparison to metestrus in both sample types (*P* = 0.0161, *P* = 0.0195 for oral and vaginal samples) (Fig. 4A).

#### Beta diversity

Oral microbiota composition analyzed by PCoA exhibited high similarities among all four estrous phases, visualized as overlaps in ordination space for both Bray-Curtis and Jaccard distances (Fig. 4B). At the same time, however, MDMR revealed significant compositional differences among the four phases (MDMR: D.F. = 3, test. stat = 5.02, *P* = 0.00538and D.F. = 3, test. stat = 6.57, *P* = 0.00193 for Jaccard and Bray-Curtis dissimilarities respectively). Looking at the dispersion of the samples in the ordination space, LMM analysis revealed significant differences among oral samples, but only for the Jaccard index (LMM: ΔD.F=3, χ^2^ = 8.2533, *P* = 0.04106). Namely, the oral samples taken in metestrus have significantly higher dispersion than diestrus samples for Jaccard distances (post hoc Tukey test: *P* = 0.042) implying higher heterogeneity of metestrus samples followed by the decrease in interindividual variability in diestrus.

PCoA for vaginal samples exhibits higher differentiation among the microbiota from different phases of the estrous cycle (MDMR: D.F. = 3, test. stat. = 5.2, *P* = 0.00138and D.F. = 3, test. stat. = 11.32, *P* < 0.0001 for Jaccard and Bray-Curtis dissimilarities, respectively). Significant differences in interindividual heterogeneity between phases of estrus were confirmed by LMM for both Jaccard and Bray-Curtis indexes (LMM: ΔD.F=3, χ^2^ = 18.506, *P* = 0.00239and ΔD.F=3, χ^2^ = 8.3129, *P* = 0.0399 for Jaccard and Bray-Curtis, respectively). Post hoc test on Bray-Curtis dissimilarities shows a significant increase of interindividual variation in estrus compared to proestrus (post hoc Tukey test: *P* = 0,05), while in Jaccard index, mice in proestrus were significantly less variable at the interindividual level than in estrus and metestrus (post hoc Tukey test: *P* = 0.0005and *P* = 0.006, respectively). Thus, in vaginal fluid, the lowest heterogeneity is detected in proestrus, while estrus and metestrus display the highest heterogeneity of microbiota (Fig. 4C). Detailed pairwise MDMR statistic for oral and vaginal samples is documented in Table S1.

#### Representation of bacterial genera during the estrous cycle

**Fig 5:**
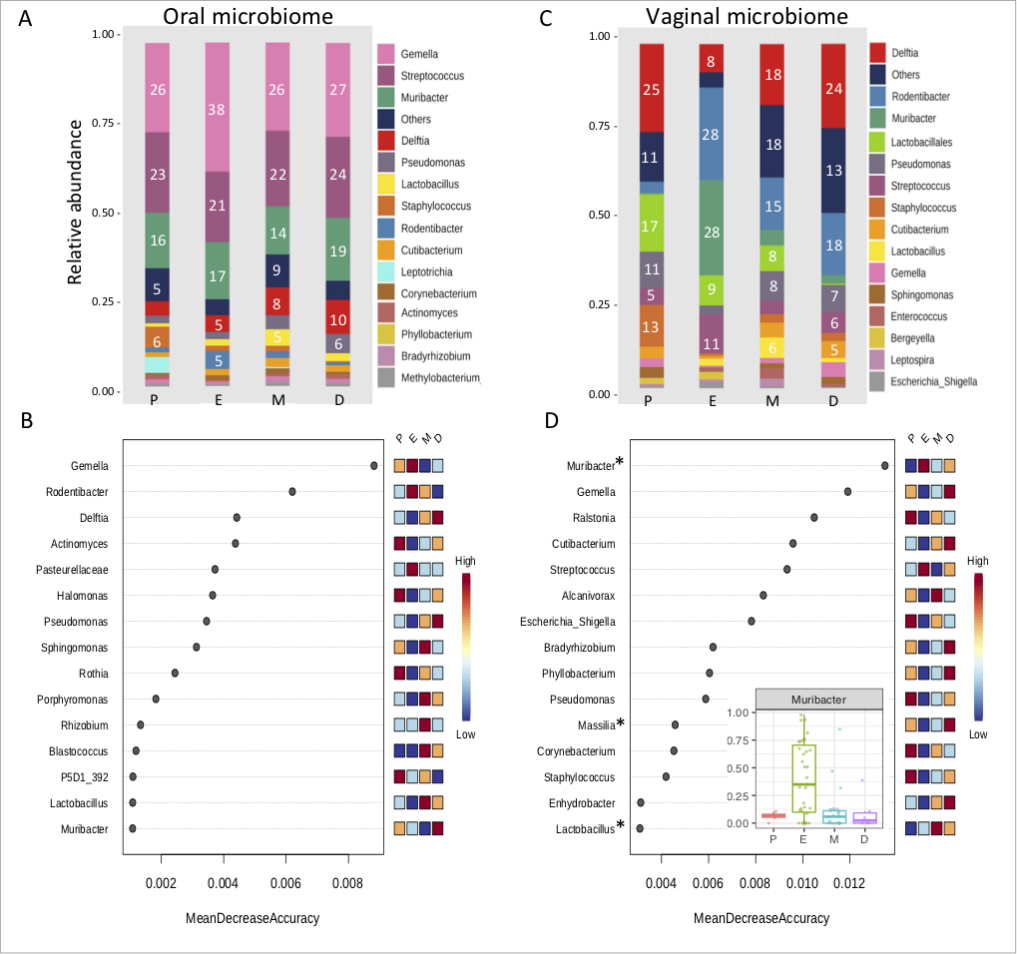
Changes in relative abundances of bacterial taxa in oral and vaginal microbiomes during the estrous cycle. Graphical visualization shows the relative average abundance of the most representative bacterial taxa in individual phases of the estrous cycle in oral (**A**) and vaginal (**C**) samples. Numbers in bar plots indicate percentage abundance. Random Forest determined bacterial genera in oral (**B**) and vaginal (**D**) samples, which are important for the classification of data into four phases of the cycle. However, in oral samples, ANCOM analysis did not confirm any significant differences in particular taxa. In vaginal samples, *Muribacter* species show the most significant increase in estrus. Significant results of ANCOM analysis are marked with a star.

In the oral microbiome, the most abundant bacterial genera do not vary during the estrous cycle (Fig. 5A). Random Forest analysis identified *Gemella* and *Rodentibacter* as the most important features for separating individual phases of the cycle in oral samples; however, the level of decreased accuracy is very low and therefore such an order has a lower informative value (Fig. 5B). ANCOM analysis of differences among the estrous phases with respect to the relative abundances of bacterial genera in oral microbiome revealed only two genera with significant changes during the estrous cycle. However, each of these two taxa, that is, *Blastococcus* and *Nocardioides* constitute on average less than 1% of the oral microbiome. Such a result strengthens the statement about the homogeneity of oral microbiota (Fig. 2A and B) that is less affected by the estrous cycle than the bacterial community in the vaginal environment.

The vaginal microbiota is dynamically changing during phases of the reproductive cycle and the receptive phase, that is, estrus, is characterized by a substantial increase in *Pasteurellaceae* species (*Muribacter and Rodentibacter*), which together form over 50% of the microbiome in estrus (Fig. 5C). The only bacterial genus with statistically significant estrus-specific abundance is *Muribacter*, which is consistently confirmed by both Random Forest analysis and ANCOM (Fig. 5D). Related *Rodentibacter* species are also highly abundant in estrus, but due to a higher variation between females, this difference was not substantially supported by ANCOM. Other significantly differentially abundant bacterial taxa are *Massila*, mostly detected in diestrus and *Lactobacillus* with the highest abundance in metestrus.

### Vaginal proteome during the estrous cycle

**Fig 6:**
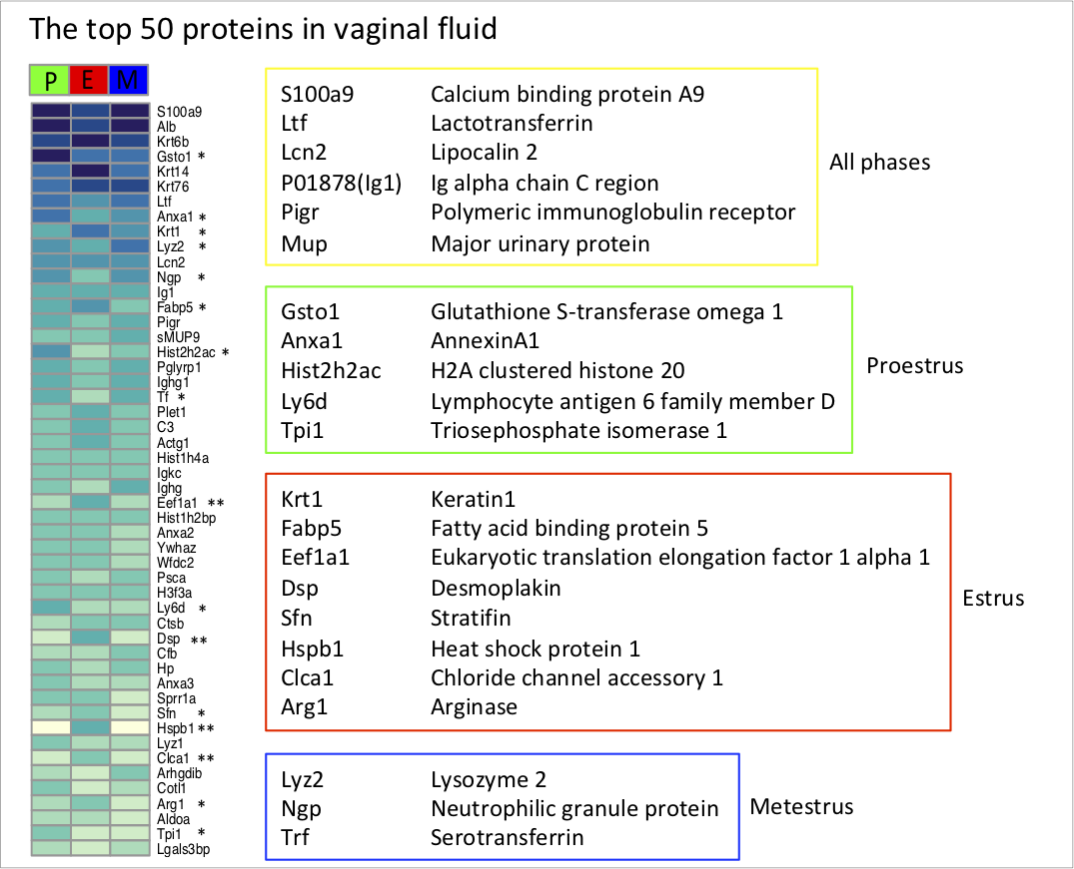
The list of the top 50 most abundant proteins in vaginal fluid. Heatmap represents the top 50 most abundant proteins in vaginal fluid. Asterisk indicates significant differential abundance during three phases of the estrous cycle, P, E, M. Next to the albumin and keratin, the most abundant proteins expressed during all phases of the estrous cycle (yellow box) are S100a9, Ltf, Lcn2, P01878, Pigr, and Mup (group of Mups similar to Mup9) which are mostly related to immune processes. Differentially abundant proteins in proestrus (green box), metestrus (red box), and metestrus (blue box) from this list are described in the boxes.

A total of 1,167mouse proteins were identified by proteomic analysis in vaginal samples. The top 50 most abundant proteins in vaginal fluids form up to 84% of the total protein volume. Along with keratins (i.e., Krt6b, Krt14, Krt7, Krt1) and albumin (Alb), there is the dominant calcium-binding protein S100a9, which represents 16% of the total protein volume. This protein has a prominent role in inflammatory processes and immune responses and increases the bactericidal activity of neutrophils (48). It is present during all phases of the cycle with a slight decrease during estrus. The top 50 list includes also other proteins involved in the defense responses, stress response, or response to toxic substances (Ltf, Lcn2, Lyz1, Lyz2, Ngp, Wfdc2, Ighg, Ighg1, Igkc, Trf, Lcp, Anxa1, Anxa2, Anxa3, Pglyrp1, Pigr, Arg1, Actg1, Ywhaz, Cotl1, Cfb, Hp, C3, Gsto1, Lcp, Glrx, Txn1, Prdx1, Prdx2, Hspb1), proteins with the capability to bind fatty acids (Fabp5) and odorant molecules (Mup—described as sMup9, i.e., similar to Mup9), and proteins involved in skin and epithelial cell development (Krt6b, Krt14, Krt7, Krt1, Dsp, Sprr1a, Sfn, Ctsb). From the group of the top 50 most abundant proteins, there were 16 proteins with differential occurrence in particular phases of the cycle: proestrus (Gsto1, Anxa1, Hist2h2ac, Ly6d, Tpi1), estrus (Krt1, Fabp5, Eef1a1, Dsp, Sfn, Hspb1, Clca1, Arg1), and metestrus (Lyz2, Ngp, Trf) (Fig. 6).

Discriminant analysis (sPLS-da) of all detected proteins revealed different proteome profiles in each of the three investigated stages (i.e., proestrus, estrus, metestrus; P, E, M). We used PLGEM statistics to identify proteins that were significantly and more than twofold differentially expressed between stages of the cycle. Some of the proteins were exclusively detected in estrus; therefore, they can be considered estrus markers related to cornification processes and protein degradation (namely Hrnr, Dsc1, Sprr1b, Dsg1a, Fmo2, Tubb2b, Tmprss11e, Psmd1, Metap2, Ppp2ca).

**Fig 7:**
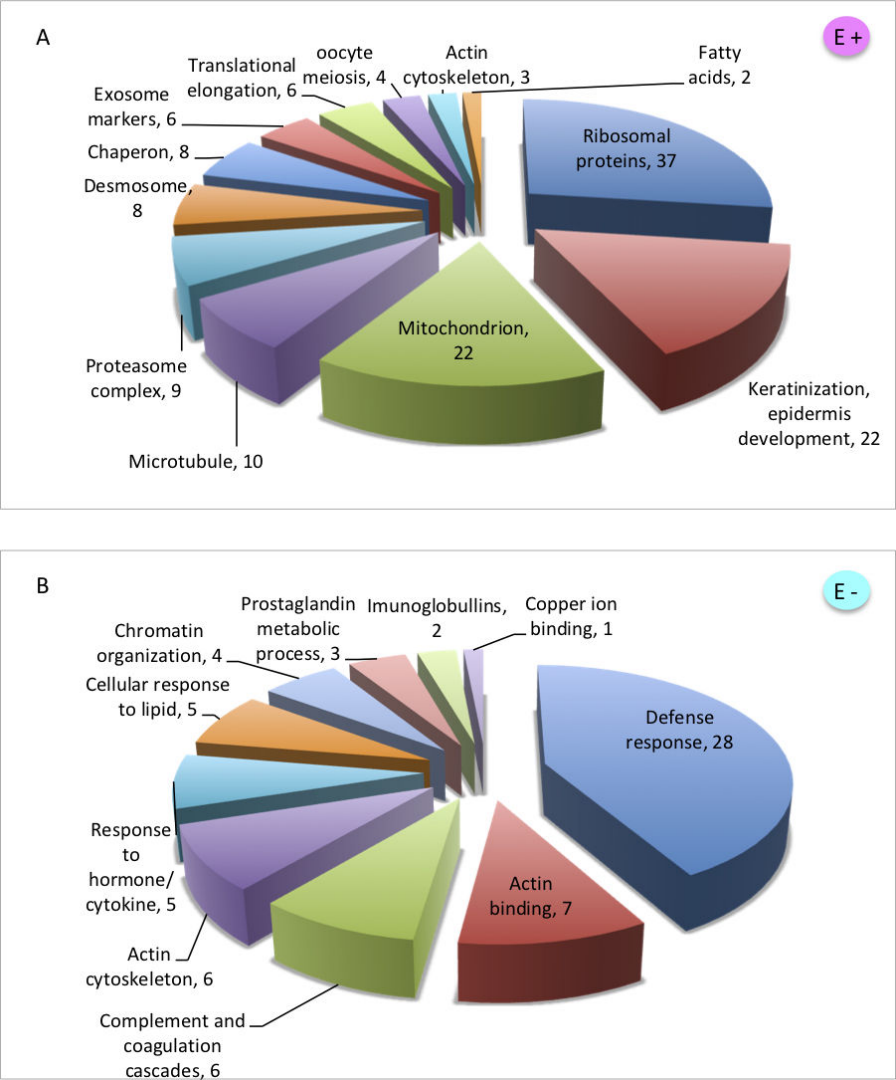
Gene ontology of proteins differentially abundant in estrus. Gene ontology analysis of 141 proteins with significantly higher abundance in estrus revealed the occurrence of mainly structural proteins associated with keratinization processes (**A**) while gene ontology of 71 proteins lowered in estrus (**B**) detected a group of defense response and immune-related proteins that decrease in estrus compared to the other two phases (i.e., proestrus, metestrus).

Furthermore, in the complete data set, we identified a total of 141 proteins more abundant in estrus (E+) and 76 proteins whose abundances were lowered in estrus (E-). To assess potential biological processes occurring in the receptive phase of the estrous cycle, we performed the gene ontology search (String-db.org), which revealed clusters of related proteins involved in various cellular processes (Fig. 10). More than 30 ribosomal proteins of small and large ribosomal subunits form the biggest cluster of estrus abundant proteins. Their presence reflects the estrus-dominant keratinization process during which degeneration of ribosomes and other cellular components occurs; therefore, they are highly detectable in extracellular space. We have also detected highly abundant proteins involved in epithelial cell differentiation, a cluster of proteasome complex proteins and proteins expressed in cellular components like desmosomes, mitochondrions, microtubules, or proteins with chaperon function (Fig. 7A).

Interestingly, the majority of proteins that were detected with lower abundances in estrus were mostly annotated as part of the immune system, defense response, or response to hormones (Fig. 7B). Some immune-related proteins decreased during estrus and increased in proestrus and metestrus (Apoa4m B2m, Cfl1, Chil3, Kng1, Prtn3, Serpina1b, Serpina1c), while others have higher abundance only in proestrus (Ahsg, Anxa1, Apoh, Chil4, Clu, Gstp1, Il1rn, Itnl1b, Ptpn6, Sftpd, Wdr1) or metestrus (Camp, Ces1c, Elane, Lyz2, Msn, Ngp, Plg, Trf) (Fig. 10). Increased antimicrobial proteins in metestrus may play important roles in the regulation of bacterial species that expanded during estrus.

### Vaginal metabolome during the estrous cycle

**Fig 8:**
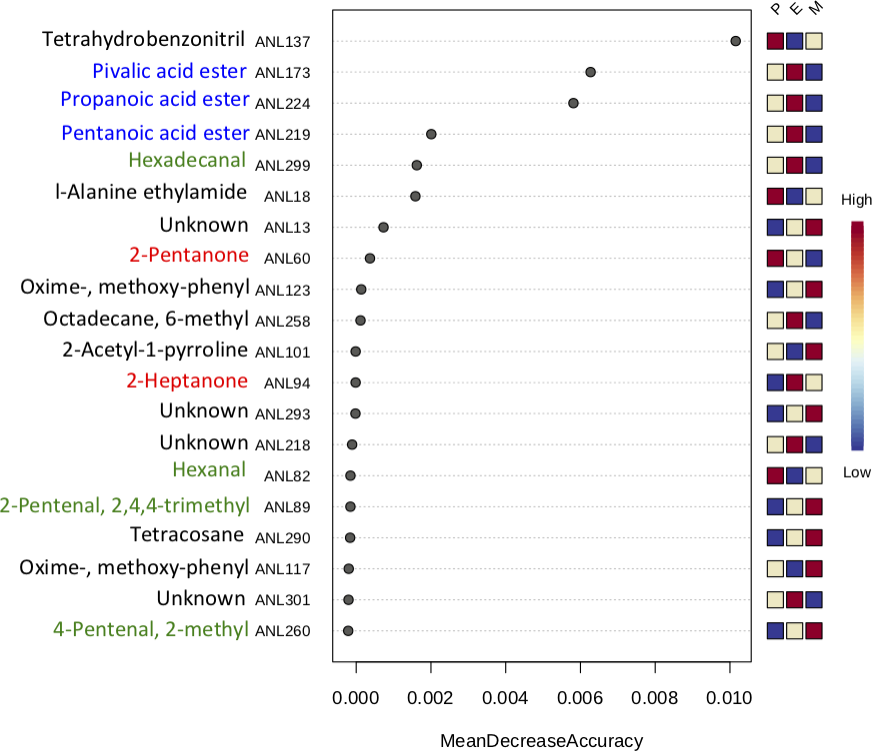
Random Forest analysis of metabolites that are important for the classification of data into three phases of estrus. Annotated compounds from the vaginal fluid belong mostly to three categories: esters of carboxylic acids (blue), aliphatic aldehydes (green), and ketones (red). Random Forest analysis implies the importance of particular metabolites to different phases of the estrous cycle; however, we did not identify any individual metabolite significantly occurring in a particular phase of the estrous cycle. This may be due to the small effect of many different substances in signaling the reproductive state of a female.

The same subset of samples as for proteomic analysis was analyzed by GCxGC-MS to identify volatile metabolites and determine their abundance during the three phases of the estrous cycle. The total of 66 metabolites (Supplementary file 1) was analyzed by discriminant analysis (sPLSda), which revealed a clear separation of samples from different phases of the estrous cycle. However, we did not detect any particular phase-specific metabolites with PLGEM statistics. Using Random Forest analysis, we display 20 most important metabolites that may be involved in phase separation (Fig. 8). The most frequently identified analytes belong to the group of esters of carboxylic acids (ANL224, ANL173, ANL219), aliphatic aldehydes (ANL299, ANL82, ANL89, ANL260), and ketones (ANL60, ANL94). Some of the molecules were previously described as pheromones in mammals, for example, 2-heptanone (49), hexadecanal (50), dimethyl disulfide (51), or acetophenone (52). Of the 20 analytes that were detected by Random Forest analysis, eight show a higher abundance in estrus (ANL173, 224, 219, 299, 258, 94, 218, 301); therefore, it is highly likely that there are mixtures of volatiles shaping a specific estrus pheromonal blend and that individual molecules have rather a small effect.

### Correlation of provided OMICs data sets

**Fig 9:**
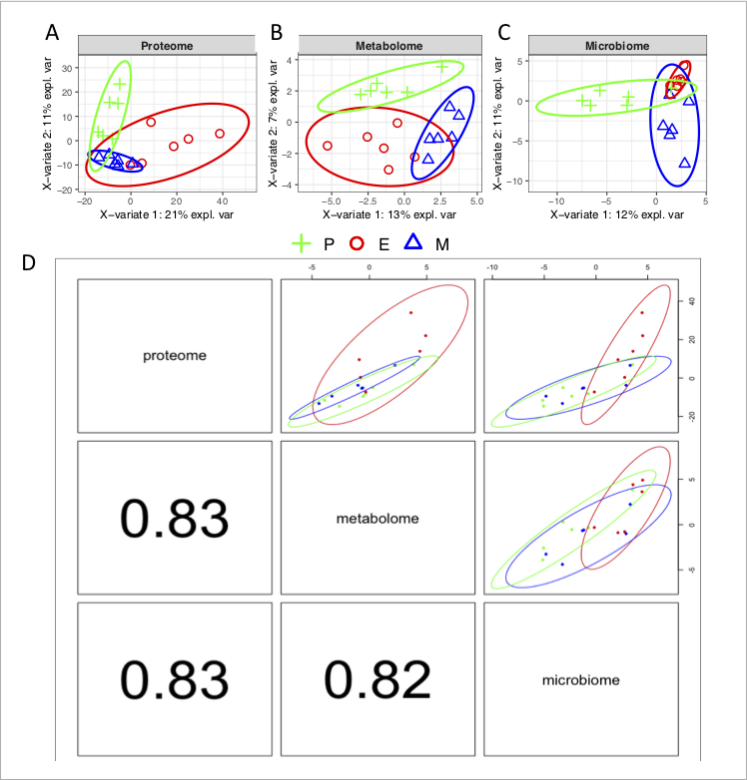
Data integration and discriminant analysis of microbiome, proteome, and metabolome from vaginal samples. Discriminant analysis (sPLS-da) of proteome (**A**) metabolome (**B**) and microbiome (**C**) data set clearly separates three phases of the estrous cycle. Data integration revealed that all three datasets (**D**) could equally differentiate among stages of the estrous cycle. High correlation coefficients support the view that microbiomes, host proteomes, and metabolomes interact.

In our recent paper, we provided evidence that volatile metabolomes and urine proteomes are correlated at the level of components (39). To find out whether there is also a potential interaction between microbiomes, proteomes, and metabolomes, we used the discriminant analysis and discriminant analysis on blocks. In Fig. 9A through C, we clearly see that particular phases of the cycle—namely proestrus, estrus, and metestrus—in all three datasets are sufficiently separated thus providing evidence that each phase of the cycle has a unique profile. Data integration further revealed that all three datasets are highly correlated (*r* > 0.8, *P* < 0.05) on the level of components. This result supports two conclusions. First, there are strong statistical interactions between microbes, host proteomes, and metabolomes. Second, all three kinds of data provide similar information on the estrous cycle at least to a human observer; however, it needs to be further elucidated on what particular molecules are responsible for olfactory detection of phase-specific odor space.

## DISCUSSION

The community of bacteria inhabiting different parts of the host body is highly specific and subject to different regulatory processes depending on the specific micro-environment and host-microbiota interaction. The oral microbiome is highly exposed to the external environment so it can be expected to show an increased bacterial diversity. The vaginal microbiome is also influenced by external factors, for example, the mating system, and it was already reported that promiscuous species in some rodents have a higher bacterial diversity (18, 53). Our results show that the oral microbiome has a higher alpha diversity in observed OTU and exhibits a higher homogeneity between samples compared to the vaginal microbiome (Fig. 2E and F). This is caused by prominent changes in microbiome composition in vaginal samples during particular phases of the estrous cycle. Furthermore, interindividual variation of microbiota composition was increased in estrus. These changes are highly likely associated with fluctuating estrogen levels and massive keratinization processes during estrus. A higher level of estrogen in the vaginal environment is followed by an increase in glycogen concentration in the vaginal mucosa (54), which serves as nutrition and enables bacterial growth. It is documented in conventionally reared animals (mice, rats, rabbits, and dogs) that bacterial populations massively invade keratinized cells during estrus (55) and that bacterial abundance is much higher in estrus than in other phases (32).

Overall, our data show that enhanced bacterial growth through improved substrate availability (i.e., keratinized cells) along with reduced host immune responses in estrus leads not only to changes in community composition but also to increased microbiota heterogeneity between individuals. This is consistent with the “Anna Karenina” principle (46), which predicts increased interindividual variation in the composition of the microbiota when host mechanisms aimed at its regulation are downregulated.

Although the keratinization process also occurs in the oral cavity and the amount of keratinocytes is higher in increased estrogen levels (56), we did not identify any particular bacterial taxa with significant differential abundance during the phases of the female reproductive cycle in saliva samples (Fig. 5A and B). Our results show that the predominant bacterial species found in the oral microbiome include *Gemella* and *Streptococcus,* which can be generally found in various laboratory mouse strains (57), while the high abundance of *Muribacter* and the low amount of *Lactobacillus* seem to be characteristic of wild mice oral microbiome.

Unlike humans, other mammalian species including primates lack the dominance of *Lactobacillus* species; however, their microbiomes show changes during the estrous cycle with distinctive bacterial communities found at the ovulation/estrus peak (12, 58). Although rodents, and specifically the house mouse, are used as model organisms for human vaginal dysbiosis or other pathologies, the description of the normal physiological state of the rodent vaginal microbiome is very scarce (19, 21, 22) and contradicting. According to Vrbanac et al. (21), the laboratory C57BL/6 mouse vaginal microbiome is dominated by *Staphylococcus* and *Enterococcus* and the fluctuation of microbiome is independent of the estrous cycle. Moreover, they did not detect any *Pasteurelaceae* family members, such as *Muribacter* or *Rodentibacter*. This discrepancy may be the outcome of an artificial selection and inbreeding of the laboratory mouse strains that led to the loss of natural sources of microbiome. The family *Pasteurellaceae*, especially the genus *Rodentibacter*, includes species with high virulence potential (59), and their presence in breeding facilities is considered undesirable according to FELASA (60). Similarly, it can be difficult to distinguish *Muribacter* from other closely related *Pasteurellaceae* pathogens (61), which may partially explain their absence in laboratory mice. However, in laboratory rats, *Proteobacteria*, *Firmicutes,* and *Actinobacteria* dominate the vaginal microbiome, and unclassified member of the *Pasteurellaceae* family belongs to the most abundant OTU. Moreover, changes in bacterial community in rat females follow the estrous cycle, with *Streptococcus* increasing during estrus, and *Corynebacterium* increasing during diestrus (22).

Dominant bacterial genera in the vaginal microbiome in wild mice are *Delftia*, *Muribacter,* and *Rodentibacter*. Especially in the estrus phase, *Muribacter* and *Rodentibacter* from *Pasteurellaceae* family constitute more than 50% of all reads (Fig. 5C). *Rodentibacter* spp. are common bacterial taxa in wild rodents; it was detected in vaginal and salivary microbiomes of four species of wood mice (*Apodemus*) (18). *Muribacter* was also detected in the oral and vaginal microbiome of wood mice; however, with low abundance, therefore this bacterial species seems to be more specific for the house mouse.

One of the recent research (6) clearly showed differences between wild and laboratory mice in many aspects, including microbiome and immune system. They generated a mouse model by transferring the embryo of laboratory mice (C57BL6) into wild mice resulting in restoring the natural microbial environment in such newborn mice (“wildling”). Interestingly, vaginal microbiome of wildlings and wild mice contained a high percentage of *Pasteurellaceae* taxa when compared to conventional laboratory mice. In wild mice, the virulence of *Rodentibacter* is considered to be low; however, intranasal infection with *Rodentibacter heylii* resulted in 100% and 75% mortality in BALB/c and C57BL/6 mice, respectively. The same dose infection with *Muribacter muris* induced an immune response but did not cause the death of animals (62). Loss of natural bacterial species may therefore influence the activity of the immune system. Nonetheless, the lethal infection could also be dose-related because in a co-housing experiment of C57BL/6 and wild mice no such effect was detected, and all mice survived a period of 3 months of co-housing (63).

The *Lactobacillus* spp. are the most common in human vaginal microbiota where they maintain an extremely acidic environment by producing lactic acid, which protects the vaginal environment by restricting the growth of pathogenic organisms (64). Some reports suggest that in the laboratory mice, *Lactobacillus* spp. are abundant species; however, these findings may be caused by the different methodologies used in their experiments (i.e., qPCR) (65). In our study, the genus *Lactobacillus* was abundant only in a few samples from our data set and was overrepresented not in vaginal microbiota as we would expect but in oral microbiota (Fig. 3B). Human vaginal acidic pH (~4.5) triggered by the excess of lactobacilli is unique in mammalian taxa. In other mammalian species including primates, the occurrence of *Lactobacillus* spp. is in the range of 1%–5% with vaginal pH varying between 6 and 7. In addition to the fact that lactic acid may be produced by other bacterial species, there are probably other mechanisms for how commensal bacteria protect the vaginal environment.

**Fig 10:**
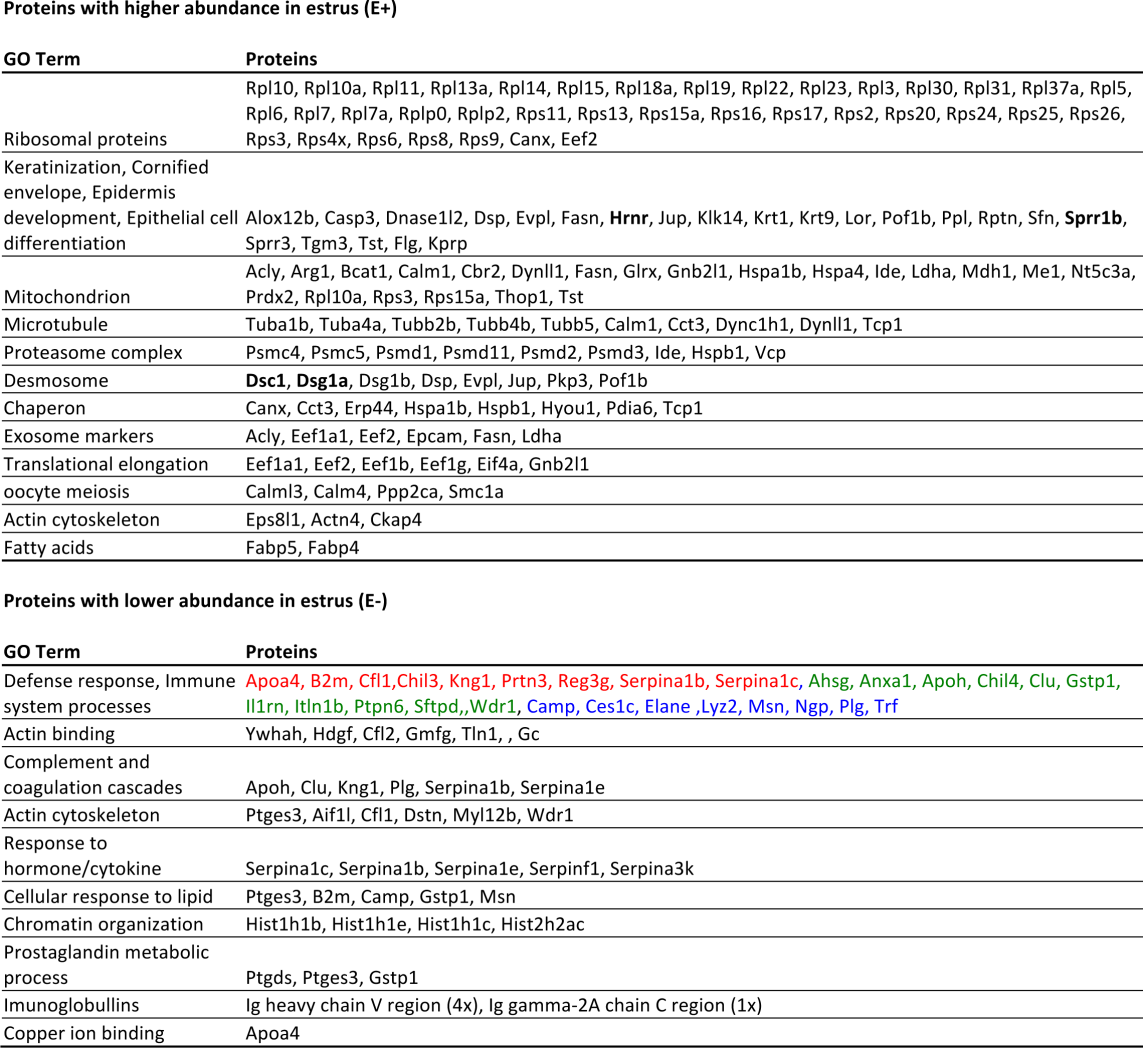
Gene ontology terms characterizing proteins that were more abundant in estrus and proteins with lower abundance in estrus. Proteins with higher abundance in estrus—Hrnr, Sprr1b, Dsc1, and Dsg1a could be considered as estrus markers as they were consistently detected only in estrus and not in other stages in all tested females. The group of proteins with lower occurrence in estrus associated with defense response are colored according to the phase of the estrus cycle where they are dominant compared to the other phases. The significant increase in proestrus and metestrus compared to estrus (red), the significant increase in proestrus (green), and the significant increase in metestrus (blue).

The most abundant protein in vaginal sample S100a9 occurs in all three stages of the cycle and it is detected as an abundant protein in the reproductive tract of various mammals (30, 66, 67) including humans (68). In many mammals, the estrus stage cell profile is characterized by the excess of keratinized cells that are heavily populated by bacterial colonies. Similarly, as in Lamy et al. (67), we have identified many intracellular proteins abundant in estrus (mainly ribosomal subunit proteins, mitochondrial proteins, and proteins involved in keratinization), which suggested that they are released during the process of keratinization after the cell death. Therefore, the most dominant markers of estrus mirror the cellular profile and are linked to keratinization, namely Hrnr, Sprr1b, Dsc1, Dsg1a; however, we detected high estrus abundance of heat shock protein family members (Hspb1, Hspa1b), which are suggested to promote the sperm viability (31) or proteins involved in fatty acid metabolism (Fabp4, Fabp5). The most interesting result of the proteomic analysis demonstrates the decrease in extensive amount of proteins linked to defense response to bacteria in estrus (Fig. 10). This may be caused by the absence of innate immune components like neutrophils, which invade the epithelium with increasing progesterone level (33) during metestrus where we detected an increase in neutrophil producing antimicrobial proteins like Camp, Lyz2, Ngp, Trf (26, 28). However, epithelial cells may also be a source of innate immune protein production. Proestrus is characteristic of excessive amounts of differentiating epithelial cells and therefore, on the protein level, we can see different types of immune-related proteins in this phase, for example, Ahsg, Anxa1, Il1rn, Itln1b. Our results suggest that there may be variable mechanisms of host-microbiome regulation in different phases of the estrous cycle.

The concept of symbiotic microorganisms influencing chemical communication in mammals, often referred to as the “fermentation hypothesis,” is not a novel one (69). However, due to recent technological advancements and the accessibility of omics methods, it is now possible to provide evidence that specific molecules serve as messengers to signal the receptive phase of the estrous cycle. We have identified some of the volatile compounds that play roles in olfactory signaling and are already known to contribute to behavioral responses (Fig. 8). Esters of carboxylic acids (i.e., propanoic, pentanoic) are widely observed as mouse body scent components, important for mice in the recognition of their conspecifics (70) and as an estrous specific chemical signal in vaginal fluids and saliva (71) in the bovine. Aliphatic aldehydes (hexadecanal, pentanal) are well-known mouse body odor components as evidenced by the presence of receptors for these aldehydes, which have been described in the main olfactory organ (72, 73). These components may play a key role for mice in recognizing and signaling their conspecifics from a distance (74, 75). One of the long-chained aliphatic aldehydes, hexadecanal, plays a role in the phenomenon called social-buffering in mice (76). Hexadecanal also participates in chemo-signaling in humans and influences their behavior, suppressing aggression in males and increasing aggression in females (50). Another typical class of volatile organic compounds that stimulate the olfactory system of mammals are aliphatic ketones and they are often found in the odor of rodent urine (77, 78). Among others, we detected 2-Pentanone, a highly abundant estrus marker in cervicovaginal mucus in bovine (79), and 2-acetyl-1-pyroline that mediates the communication of the binturong signature scent and conveys information about sex and reproductive state (80). In humans, 2-acetyl-1-pyroline was detected as one of the potential intravaginal odor-active compounds that may play a role in sperm chemotaxis (81). 2-heptanone is part of the mouse urine and it is an active substance that participates in puberty delay in females (82) and it was also described as an alarm pheromone in various insect species (83). Acetophenone is produced predominantly by skin microbiota and the emission of this molecule was found to be higher in flaviviruses infected individuals, which, in turn, makes their odor attractive to mosquitos (52).

It is clear that complex olfactory information is not based on a single substance but is rather based on a proportional mixture of different substances also called a pheromonal blend (84, 85). The reason why we could not identify any particular metabolite significantly abundant in estrus may be therefore due to the small effect of many different compounds in signaling the female reproductive state.

### Conclusions

Our results contribute to a deeper understanding of the complex relationship between the mammalian host proteome, its microbiome, and the formation of chemical signals that serve as olfactory cues. We reported two different microbiome systems of an individual, oral and vaginal, and showed that the dynamic changes of bacterial communities are not dependent only on hormonal fluctuation but it is probably the change in cellular composition that forces the dynamic changes of microbiome. The extent to which chemical signals produced by the microbiome are species specific or influenced by substances found in the environment needs to be further studied.

## MATERIALS AND METHODS

### Animals and sample collection

Between November 2018 and January 2019, a total of 18 females of *Mus musculus musculus* mice were lived-trapped at three farms in central Bohemia [Brandýs nad Labem (50.187N, 14.667E), Písnice (49.991N, 14.464E), and Skupá (50.017N, 13.722E)], taken to the animal facility at Charles University, Prague and caged separately in standard plastic cages (275 × 215 x 140 mm) with a wire mesh lid and with *ad libitum* access to water and food (ST1 food, Velaz, Prague, Czechia). Animals were caged in the mouse facility for 4–12 weeks before the experiment started. All females selected for the experiment were adults, sexually mature, and of approximately the same size. During the experiment, samples of oral and vaginal microbiota were collected daily at the same time (morning) for 7 days in a row, then the mice were left for 2 weeks without sampling, followed by another 7 days in a row of sampling. Oral and vaginal samples were noninvasively collected by gentle flushing with 2 × 30 µL of sterile dH2O. 5 µL of the sample was used for cytological analysis using standard protocol to visualize nucleated and cornified cells with May-Gruenwald (3 min) and Giemsa (10 min) staining solutions (8). All samples were immediately frozen at −20°C. To avoid bacterial contamination, sterile pipette tips with filters were used for sample collection, and mice were handled using disposable laboratory gloves. One microtube was filled with 2 × 30 µL of sterile dH2O and kept open during each day of sampling to control potential contamination from the surrounding environment.

### Preparing the library

DNA was extracted from the 30 uL of each sample using Quick-DNA Fungal/Bacterial Microprep Kit (catalog no. D6007, Zymo Research), according to the manufacturer’s protocol. In the final step, we eluted DNA from the spin columns using a total of 30 µL of the kit’s elution buffer. One microtube from each kit was processed without adding a sample to serve as a control of potential contamination from the kit.

For 16S library preparation, we used primers to amplify the V3-V4 region of the 16S rRNA, forward: S-D-Bact-0343-a-S-15F TACGGRAGGCAGCAG and reverse: S-D-Bact-0785-a-A-21R ACTACHVGGGTATCTAATCC. Both primers were provided with specific oligonucleotide sequences compatible with Illumina sequencing adaptors in the following second PCR. For each of the first PCRs, we used 5 µL of KAPA HIFI Hot Start Ready Mix (Kapa Biosystems, USA), 1 µL of F and R primer mixture (5 µM) and 4 µL of DNA template. The PCR conditions were as follows: initial denaturation at 98°C for 2  min, followed by 35 cycles each of 98°C (20  s), 55°C (30  s) and 72°C (30  s) and a final extension at 72°C (5  min). PCR products were purified with AMPure XP beads (Beckman Coulter) to eliminate free primers and primer dimer species.

The second PCR step (eight cycles) to attach dual indices and Illumina sequencing adapters (Index PCR) was performed using the Nextera XT Index Kit with supplied Nextera XT Index Primers and we used the recommended protocol from Illumina (https://support.illumina.com/documents/documentation/chemistry_documentation/16s/16s-metagenomic-library-prep-guide-15044223-b.pdf). After purifying the final PCR products, samples were pooled according to concentration into four tubes and the quality of each library was assessed using Bioanalyzer with High Sensitivity DNA Assay. According to the results from the DNA assay (i.e., quality control) and the concentration we proceeded with the final pooling of the library. We performed qPCR library quantification of the final library pool using the Kapa Library Quantification Kit (Roche). The library was sequenced on the Illumina MiSeq platform at the Sequencing Core Facility at BIOCEV (Vestec, Czech Republic).

### Microbiome data analysis

To demultiplex samples, detect, and trim away gene-specific primers, a skewer (86) was used. In the next step, using dada2 (87) low-quality reads (expected error rate per paired-end read > 2) were eliminated and quality-filtered reads were denoised. The resulting 16S rRNA haplotypes were clustered with vsearch (88) into 97% sequence similarity OTUs. Next, UCHIME (89) was employed for the detection of chimeric OTUs, with gold.fasta (available at: https://drive5.com/uchime/gold.fa) as a reference database. The SILVA database version 132 (90) along with RDP classifier was used for bacterial OTU annotation. OTU sequences, their taxonomic annotations, and OTU table (i.e., OTU read counts in individual samples) along with sample metadata were merged into one database using the package phyloseq (91) in R (R Core Team 2015). We eliminated all OTUs that were not assigned to any bacterial phylum, or those that were of chloroplast or mitochondrial origin. Using the R package Decontam (92), we compared microbiota profiles of biological samples with 10 negative controls (blank DNA isolates). In all, 18 OTUs whose prevalence was significantly increased in the negative controls were considered putative contaminants and excluded from further analyses. Finally, we excluded two samples whose sequence coverage was lower than 1,000. The final data set for further analysis consists of 75 oral and 73 vaginal samples and comprised 2,523,935 sequences assigned to 2,069 OTUs with a mean number of sequences per sample being 16,826 (range = 3,006–95,307).

We rarefied the OTU table to achieve even sequencing depth per sample and used the down-sampled (rarefaction threshold = 3,006 sequences per sample) database for further statistical analysis. We measured the within-host microbial diversity (α-diversity) using the Shannon index and the number of OTUs (Observed). For both indexes, we then used LMMs to compare alpha diversity between oral vs vaginal microbiota and to test for the effects of the phase of the cycle (i.e., proestrus, estrus, metestrus, diestrus) with the effect of individual females processed as a random effect. The number of observed OTUs was log_10_-transformed to achieve the normal distribution of residuals. Pairwise differences in community composition between hosts (β-diversity) were analyzed using the binary Jaccard distance calculated based on OTU presence/absence and Bray-Curtis dissimilarities calculated based on the relative abundance of OTUs, Bray-Curtis is, therefore, less sensitive to compositional variation due to rare OTUs. Bray-Curtis and Jaccard dissimilarities were subsequently used to examine variation in microbiota composition using PCoA and Multivariate Distance Matrix Regression (MDMR) (93). Individual identity was considered as a random effect in MDMR models. To analyze differences in interindividual variation (i.e., dispersion) of microbiota content between vaginal vs oral microbiota and across the estrous cycle, we calculate distances to the group-specific centroid for each sample using Betadisper function (R package vegan) and used them as a response in LMMs.

We also searched for specific OTUs whose changes in abundance played a role in the compositional variation, using analysis of composition of microbiomes (ANCOM) (94) with the same model structure as described above. W statistic >0.7 was considered as a significance threshold in the case of ANCOM analyses. Figures were generated in R software, and Random Forest analysis plots were generated by the Microbiome analyst tool (95).

### Proteome and metabolome sample selection

Out of all vaginal samples whose microbiota were analyzed in this study, we selected 18 samples for additional mouse proteome and complete metabolome analyses. The selected subset of samples consists of three estrous phases (proestrus, estrus, and metestrus) from 6 females. In total, 15 µL of samples was used for the nLC-MS/MS analyses of proteins (see below) and 15 µL of these samples was measured with GCxGC-MS/MS in parallel.

### Proteome samples preparation

All protein samples were precipitated with the ice-cold acetone and then centrifuged at 14,000 rcf for 10 min at 0°C. Subsequently, a re-suspension of dried pellets in the digestion buffer (1% SDC, 100 mM TEAB – pH = 8.5) was performed. The protein concentration of each lysate was determined using the BCA assay kit (Fisher Scientific). Cysteines in 20 µg of proteins were reduced with a final concentration of 5 mM TCEP (60°C for 60 min) and blocked with 10 mM MMTS (i.e., S-methyl methanethiosulfonate, 10 min room temperature). Trypsin (1 ug of trypsin per sample) was used for the cleavage of samples at 37°C overnight. A Michrom C18 column was used for peptide desalination. Nano Reversed phase columns were used (EASY-Spray column, 50 cm x 75 µm ID, PepMap C18, 2 µm particles, 100 Å pore size). The composition of all buffers was previously described in reference (96). Eluting peptide cations were ionized by electrospraying and analyzed on a Thermo Orbitrap Fusion (Q-OT501 qIT, Thermo) with the following parameters [also described in references (96–98)]. Survey scans of peptide precursors from 400 to 1,600 m/z were performed at 120K resolution (at 200 m/z) with a 5 × 105 ion count target. Tandem MS was performed by isolation at 1.5 Th with the quadrupole, HCD fragmentation with normalized collision energy of 30, and rapid scan MS analysis in the ion trap. The MS2 ion count target was set to 104 and the max injection time was 35 ms. Only those precursors with charge states 2–6 were sampled for MS2. The dynamic exclusion duration was set to 45 s with a 10 ppm tolerance around the selected precursor and its isotopes. Monoisotopic precursor selection was turned on. The instrument was run in top speed mode with 2 s cycles.

### Metabolome samples preparation

A portion of 15 µL of vaginal lavage samples were placed in a 10 mL glass vial. The emitted volatiles were sampled using Headspace Solid Phase Micro Extraction (HS SPME) on fiber (DVB/CAR/PDMS_grey; Supelco, USA). The samples were incubated for 10 min at 50°C prior to the extraction. The extraction was carried out for 20 min. The volatiles were analyzed using the method of two-dimensional comprehensive gas chromatography with mass detection (GCxGC-MS; Pegasus 4D, Leco Corporation, USA). The system was equipped with a robotic injection system (MPS, Gerstel, Germany).

A combination of mid-polar and non-polar separation columns was used for the separation: Primary column: Rxi-5Sil MS (27 m × 0.25 mm, Restek, USA); Secondary column BPX-MS (1.4 m × 0.100 mm, SGE, Australia). Other parameters were set as follows: inlet temperature 240°C, spitless mode, constant He flow 1 mL/min, modulation time 3 s (hot pulse 0.8 s), modulation temperature offset with respect to the secondary oven 15°C. Temperature program applied on the primary oven: 35°C (hold 1 min), then increase (5°C /min) to 150°C. and the final increase (5°C /min) to 150°C (hold 3 min). The temperature offset applied on the secondary column was +5°C. Transferline temperature was held on 280°C.

The mass detector was equipped with an EI ion source and TOF analyzer enabling the unite resolution. The scanned mass range was 29–700 m/z. The ion source chamber was held at 250°C.

LECO’s ChromaTOF v4.72 was employed to control the instrument and for data processing. The mass spectra of the selected compounds were compared with those listed in a commercial mass spectra library (NIST MS 2.2, USA). Retention indices were determined using linear hydrocarbons and compared with the retention indexes in the NIST mass databases if available.

### Proteome and metabolome analyses

MaxQuant software (version 1.6.34) was used for preprocessing LC-MS data (99). For both proteins and peptides, the false discovery rate (FDR) was set to 1%, and a minimum peptide length was specified to seven amino acids. The Andromeda search engine was used for the MS/MS spectra mapping against our modified Uniprot *Mus musculus* database (downloaded in June 2015), which contains 44,900 entries. We modified our databases such that all MUP (Major urinary protein) and OBP (Odorant binding protein) sequences were removed and instead of them we have added a complete list of MUPs from the Ensembl database, and OBPs from NCBI (100). Next, we added some Tremble sequences that were missing in Uniprot, for example, KLKs, BPIs, SPINKs, SCGB/ABPs, and LCNs. Enzyme specificity was set as C-terminal to Arg and Lys, also allowing cleavage at proline bonds (101) and a maximum of two missed cleavages. Dithiomethylation of cysteine was selected as the fixed modification and N-terminal protein acetylation and methionine oxidation as variable modifications. The “match between runs” feature of MaxQuant was used to transfer identifications to other LC-MS/MS runs based on their masses and retention time (maximum deviation 0.7 min). Quantifications were performed using the label-free algorithms (99) with a combination of unique and razor peptides. All protein identifications that were identified but not quantified (zero abundances), all proteins containing less than two unique peptides, and all contaminants detected in MaxQuant output files (porcine trypsin, bovine albumins, human keratins, etc.) were removed. Following this data reduction, Quantile normalization from the “normalizerDE” package was performed (102). The final data set consists of 1167 identified proteins. For detected proteins, we use corresponding gene names in the text and figures.

All consequent analyses were performed in R software (103). To check that the data distribution conforms to the same type of distribution after normalization, we used mixtools (104). Second, we used the Power Law Global Error Model—PLGEM (105) to detect differentially expressed/abundant proteins using the functions plgem.fit and plgem-stn (104). To detect the importance of significant proteins or metabolites in the separation between phases of the cycle, we used Random Forest for Classification (106) within the R software. All plots and figures were generated in R using ggplot2 (107).

## Data Availability

Sequencing data are available at the European Nucleotide Archive under project accession number: PRJEB61883. Accession numbers for each sample are included in Table S2. The mass spectrometry proteomics data have been deposited to the ProteomeXchange Consortium via the PRIDE (108) partner repository with the dataset identifier PXD039239. Metabolomics data have been deposited to the EMBL-EBI MetaboLights database (DOI: 10.1093/nar/gkz1019, PMID:31691833) with the identifier MTBLS7748. The complete dataset can be accessed here https://www.ebi.ac.uk/metabolights/MTBLS7748.

## SUPPLEMENTAL MATERIAL

Supplemental file S1 spectrum.02037-23-s0001.xlsx Data sets of microbiome, proteome, and metabolome.

Supplemental file S2 spectrum.02037-23-s0002.pdf Mass spectra of selected analytes.

Table S1 spectrum.02037-23-s0003.xlsx Pairwise MDMR statistic for oral and vaginal samples.

Table S2 spectrum.02037-23-s0004.xlsx Microbiome analysis: accession numbers for each sample.

ASM does not own the copyrights to Supplemental Material that may be linked to, or accessed through, an article. The authors have granted ASM a non-exclusive, world-wide license to publish the Supplemental Material files. Please contact the corresponding author directly for reuse.
