# Supplementary material for: Microbial, proteomic, and metabolomic profiling of the estrous cycle in wild house mice
Source: Microbiol Spectr. 2024 Jan 3;12(2):e02037-23. doi: 10.1128/spectrum.02037-23 (PMC10846187; doi:10.1128/spectrum.02037-23)

Supplementary file2 – Mass spectra of selected metabolites.

The volatiles were analyzed using two-dimensional comprehensive gas chromatography with mass detection (GCxGC-MS; Pegasus 4D, Leco Corporation, USA). Selected spectra correspond to the volatiles listed in Figure 8.

1,2,5,6-TETRAHYDROBENZONITRILE; 1-CYANO-3-CYCLOHEXENE; 3-CYCLOHEXENE-1-CARBONITRILE; 3-CYCLOHEXENECARBONITRILE

ANL137 Similarity: 87.2% RI: 1024

Peak True - sample "10, 12ul, 10ml lahvicky, inc10min, extr20min, temp50C; splitless, headspaceSPMEgrey:1", peak 142, at 935 , 2.340 sec , sec

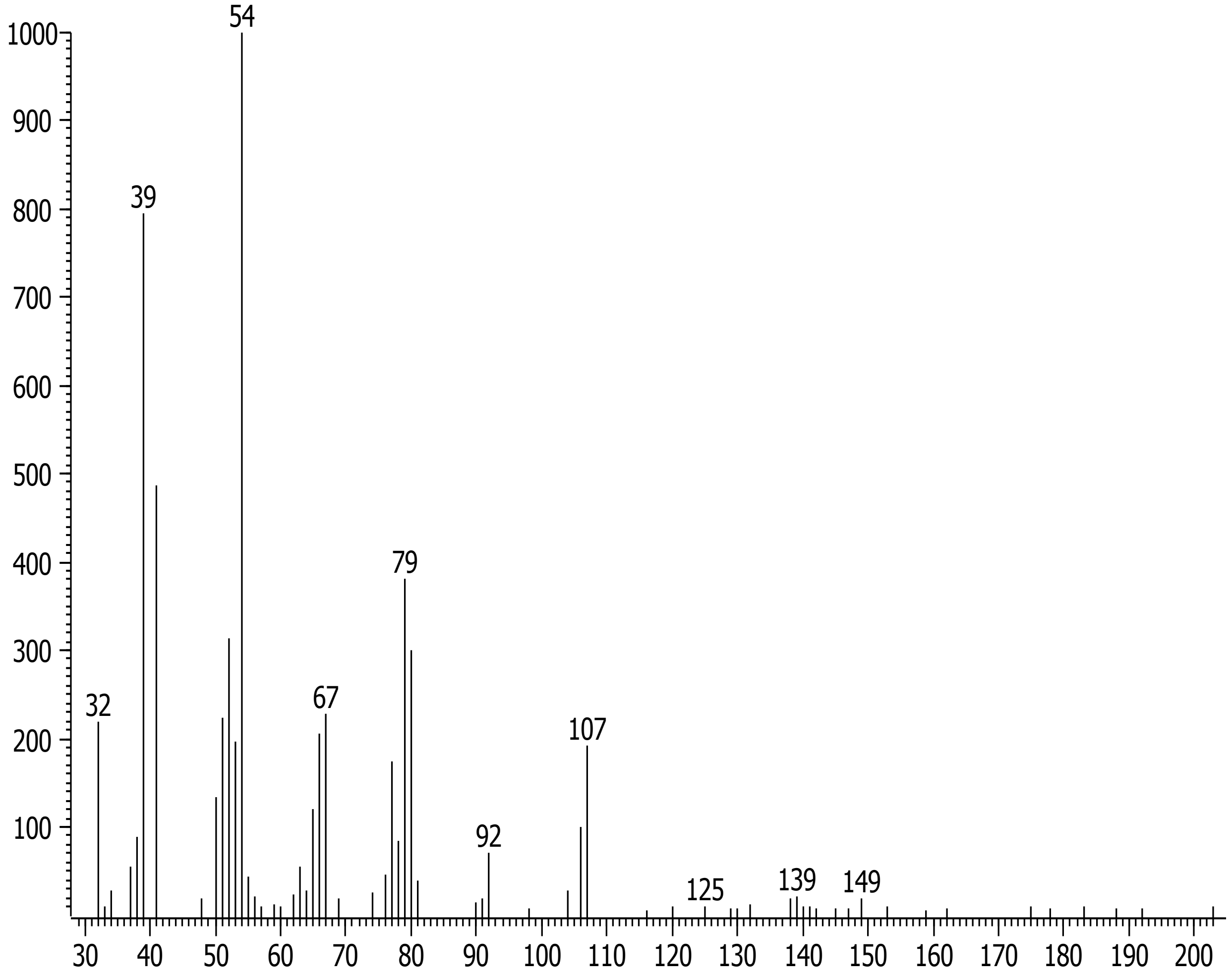

Pivalic acid vinyl ester

ANL173 Similarity: 81.1% RI: 1219

Peak True - sample "10, 12ul, 10ml lahvicky, inc10min, extr20min, temp50C; splitless, headspaceSPMEgrey:1", peak 172, at 1283 , 1.740 sec , sec

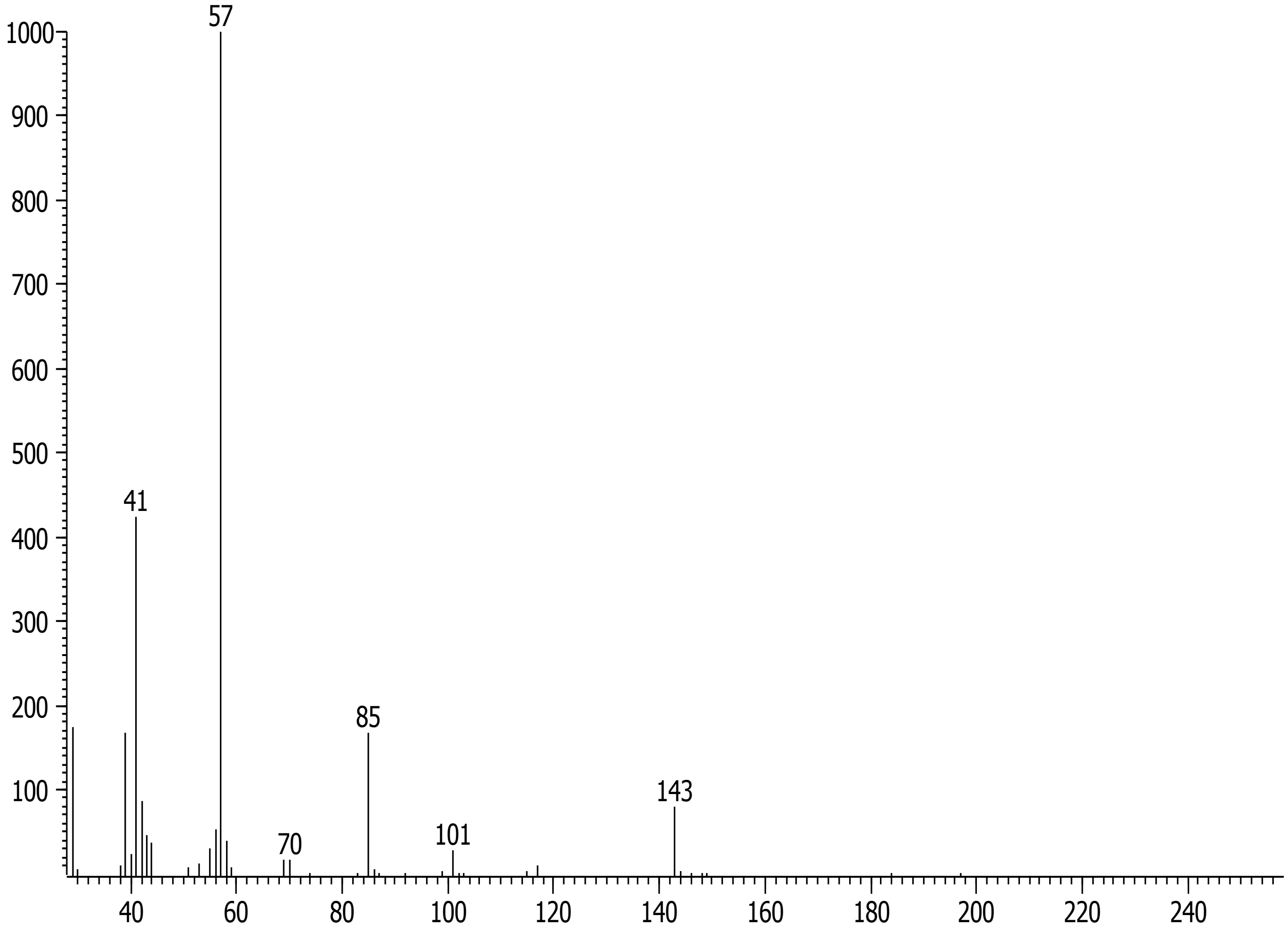

Propanoic acid, 2-methyl-, 3-hydroxy-2,2,4-trimethylpentyl ester

ANL224 Similarity: 77.7% RI: 1380

Peak True - sample "10, 12ul, 10ml lahvicky, inc10min, extr20min, temp50C; splitless, headspaceSPMEgrey:1", peak 215, at 1535 , 1.510 sec , sec

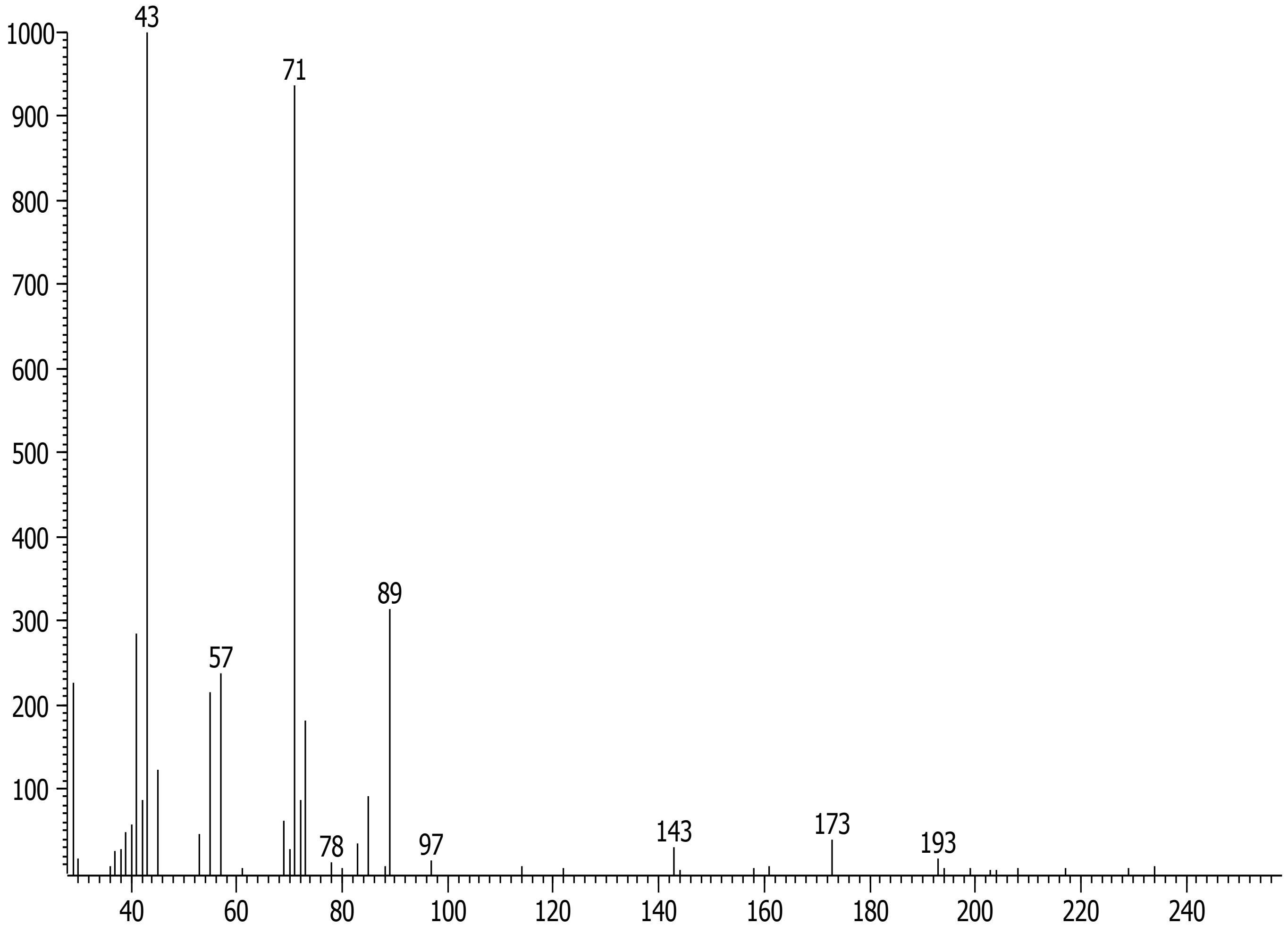

Pentanoic acid, 2,2,4-trimethyl-3-hydroxy-, isobutyl ester

ANL219 Similarity: 57.5% RI: 1361

Peak True - sample "7, 15ul, 10ml lahvicky, inc10min, extr20min, temp50C; splitless, headspaceSPMEgrey:1", peak 203, at 1508 , 1.5
30 sec , sec

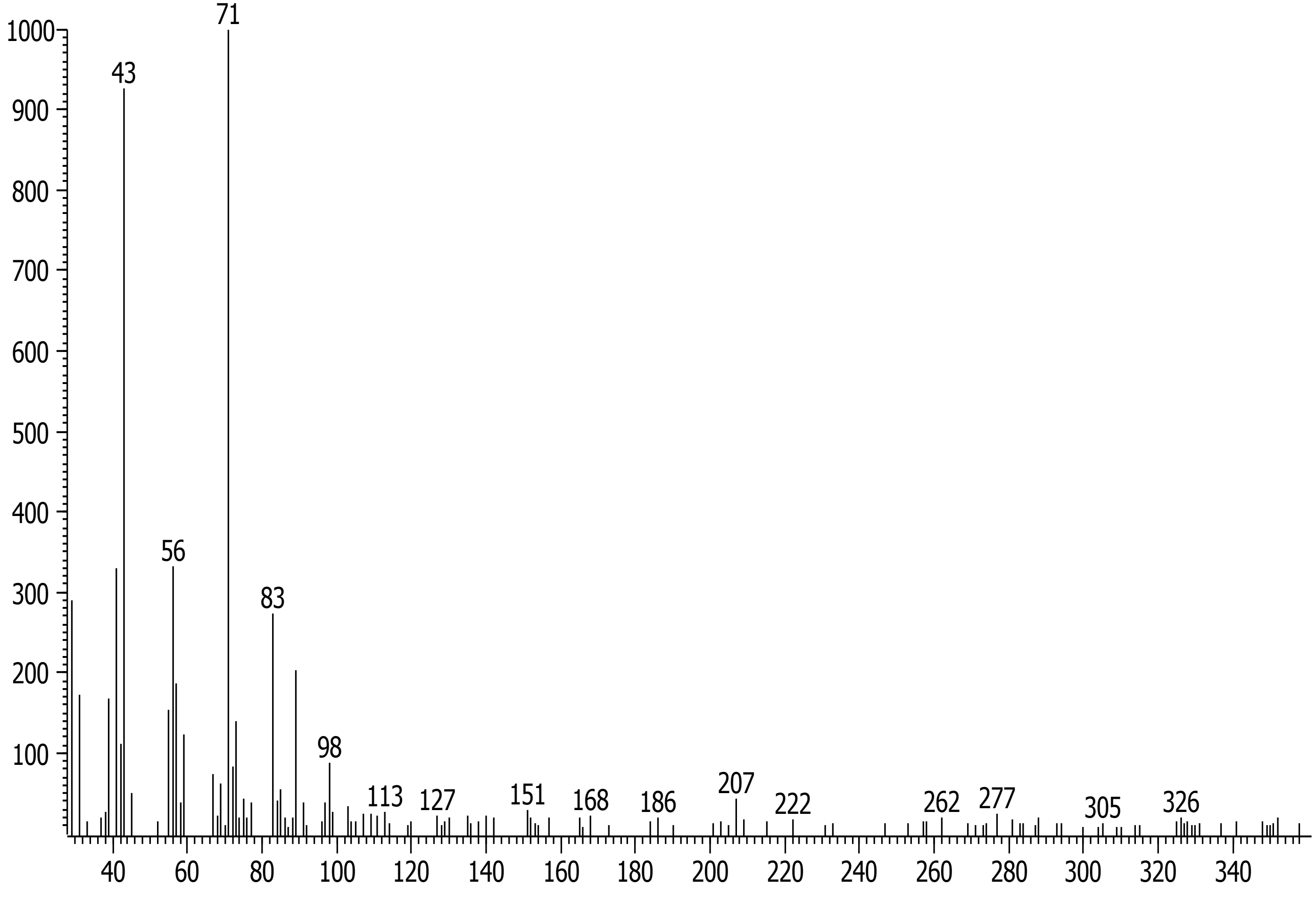

Hexadecanal

ANL299 Similarity: 55.5% RI: 1822

Peak True - sample "10, 12ul, 10ml lahvicky, inc10min, extr20min, temp50C; splitless, headspaceSPMEgrey:1", peak 287, at 1937 , 1.260 sec , sec

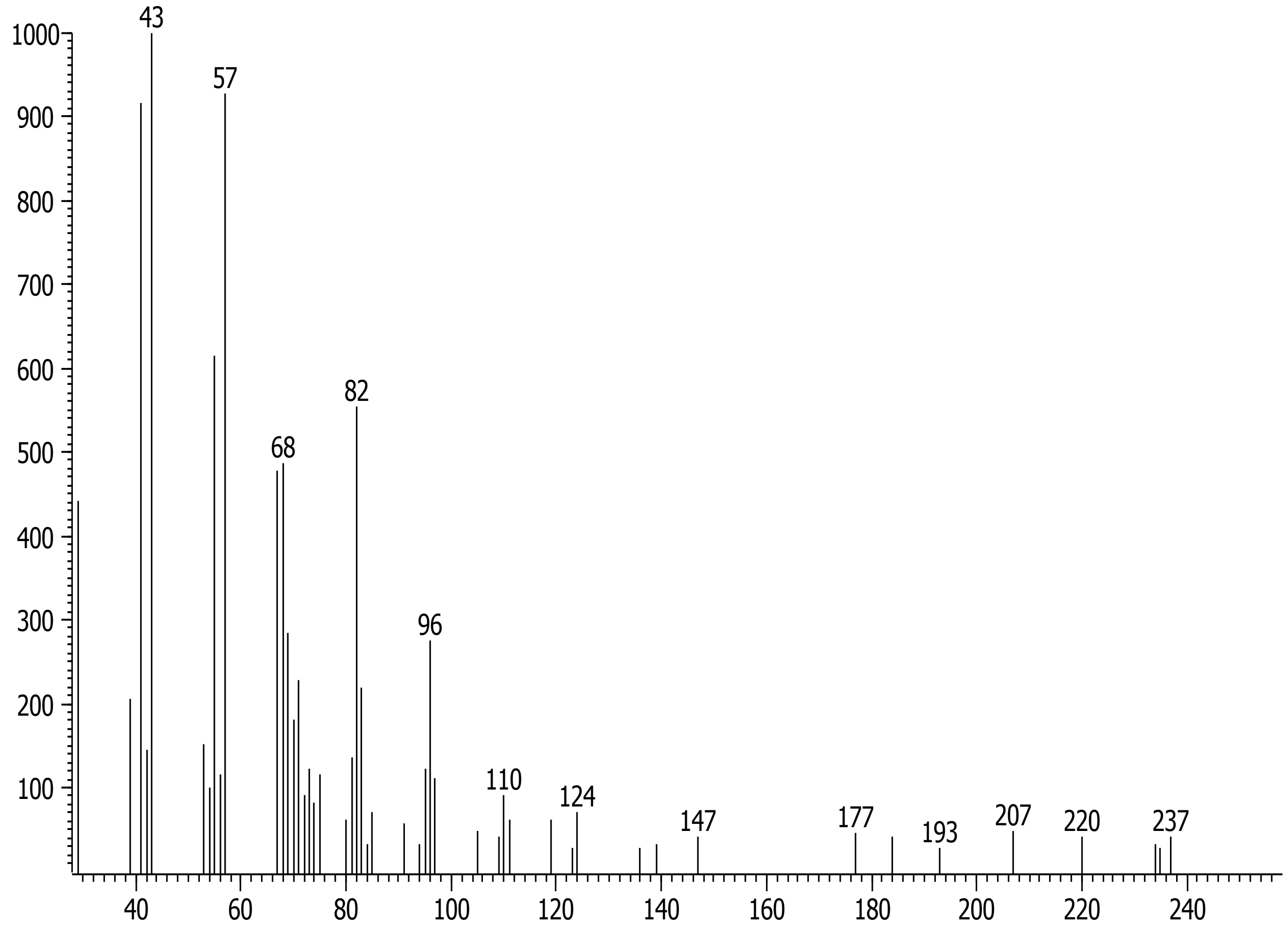

l-Alanine ethylamide

ANL18 Similarity: 97.9% RI: 649

Peak True - sample "7, 15ul, 10ml lahvicky, inc10min, extr20min, temp50C; splitless, headspaceSPMEgrey:1", peak 23, at 233 , 2.440 sec , sec

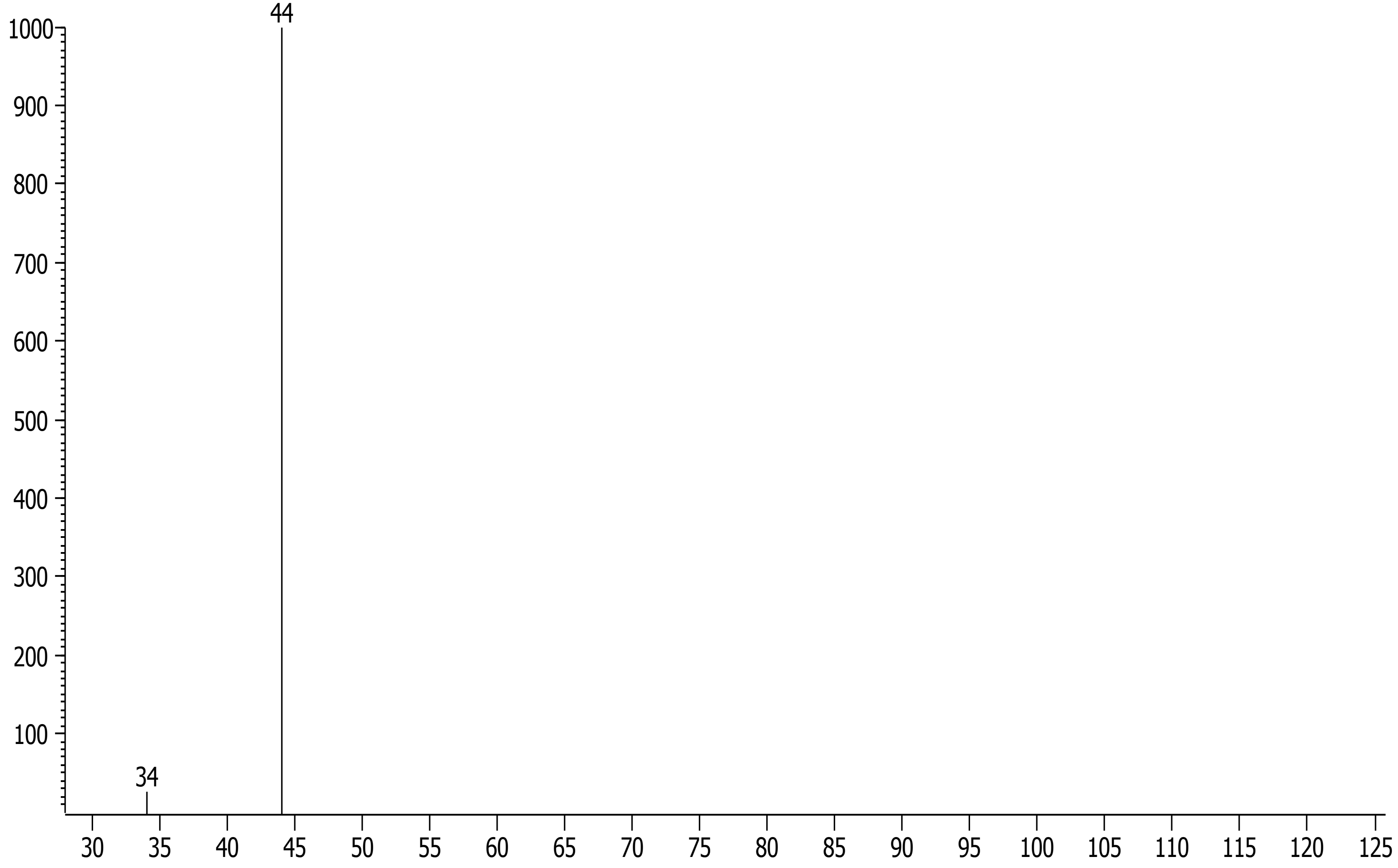

Analyte 13

ANL13 Similarity: RI: 642

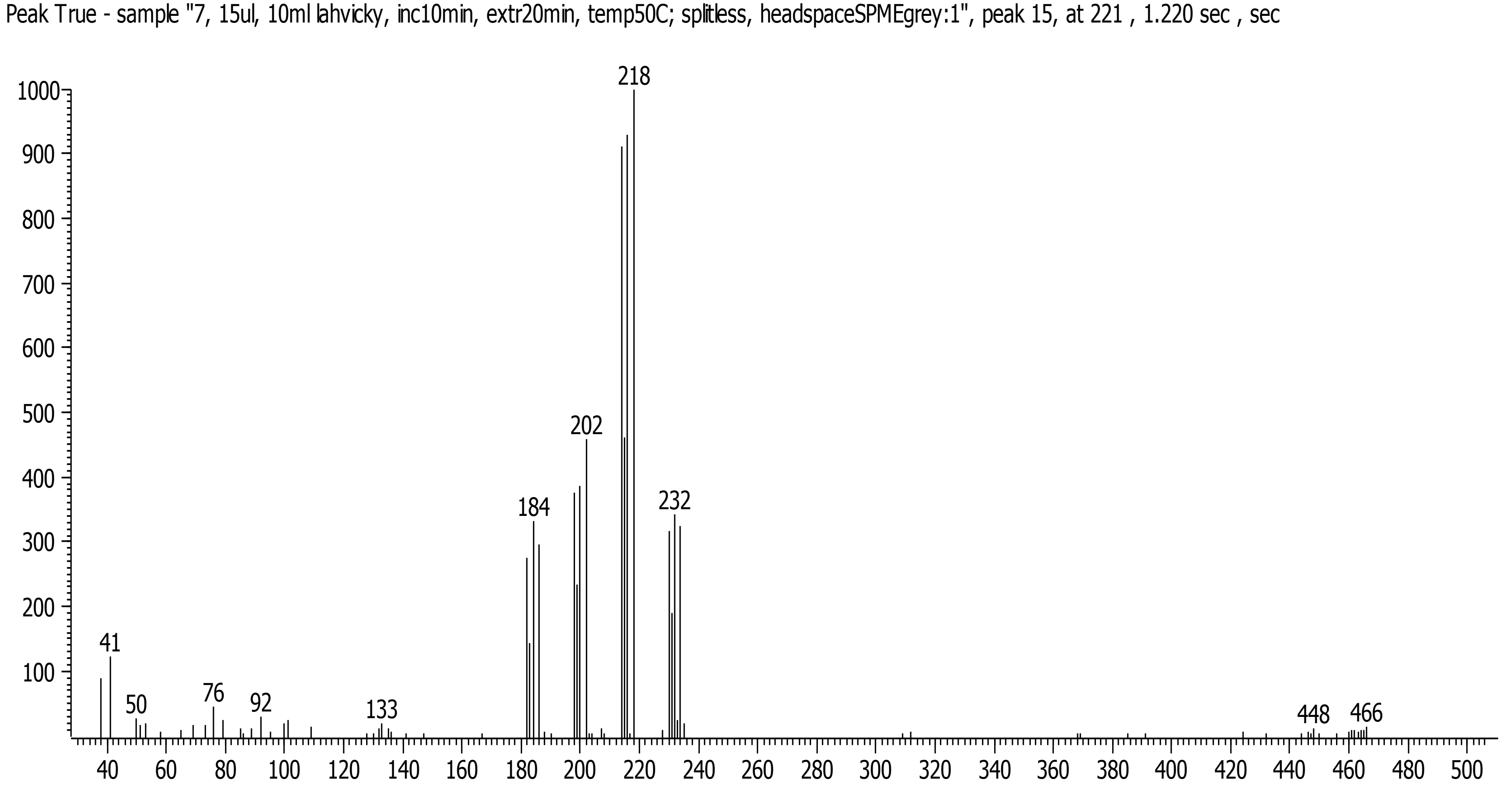

2-PENTANONE

ANL60 Similarity: 93.1% RI: 712

Peak True - sample "4, 15ul, 10ml lahvicky, inc10min, extr20min, temp50C; splitless, headspaceSPMEgrey:1", peak 74, at 359 , 1.330 sec , sec

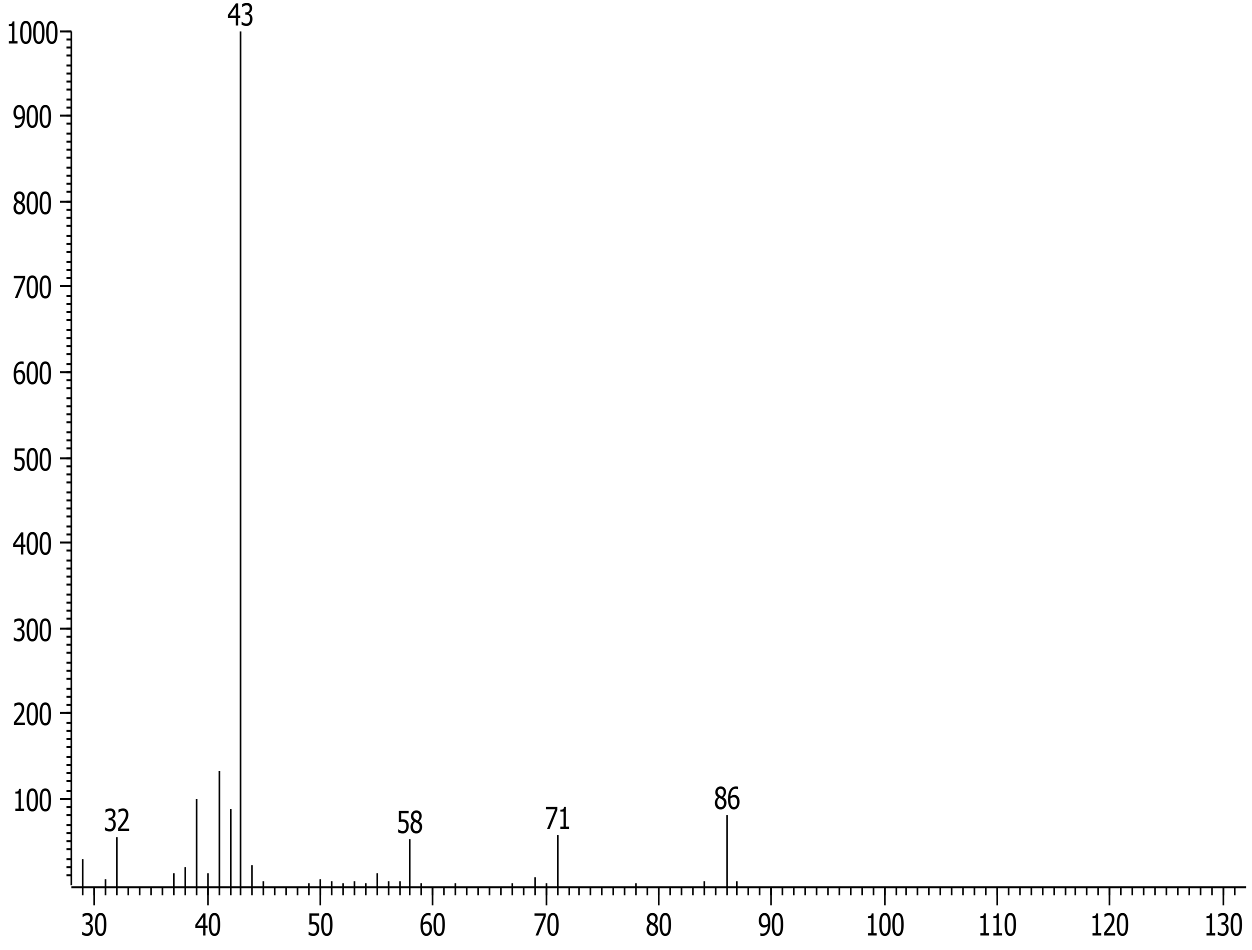

Oxime-, methoxy-phenyl-_

ANL123 Similarity: 79% RI: 986

Peak True - sample "5, 15ul, 10ml lahvicky, inc10min, extr20min, temp50C; splitless, headspaceSPMEgrey:1", peak 131, at 869 , 1.250 sec , sec

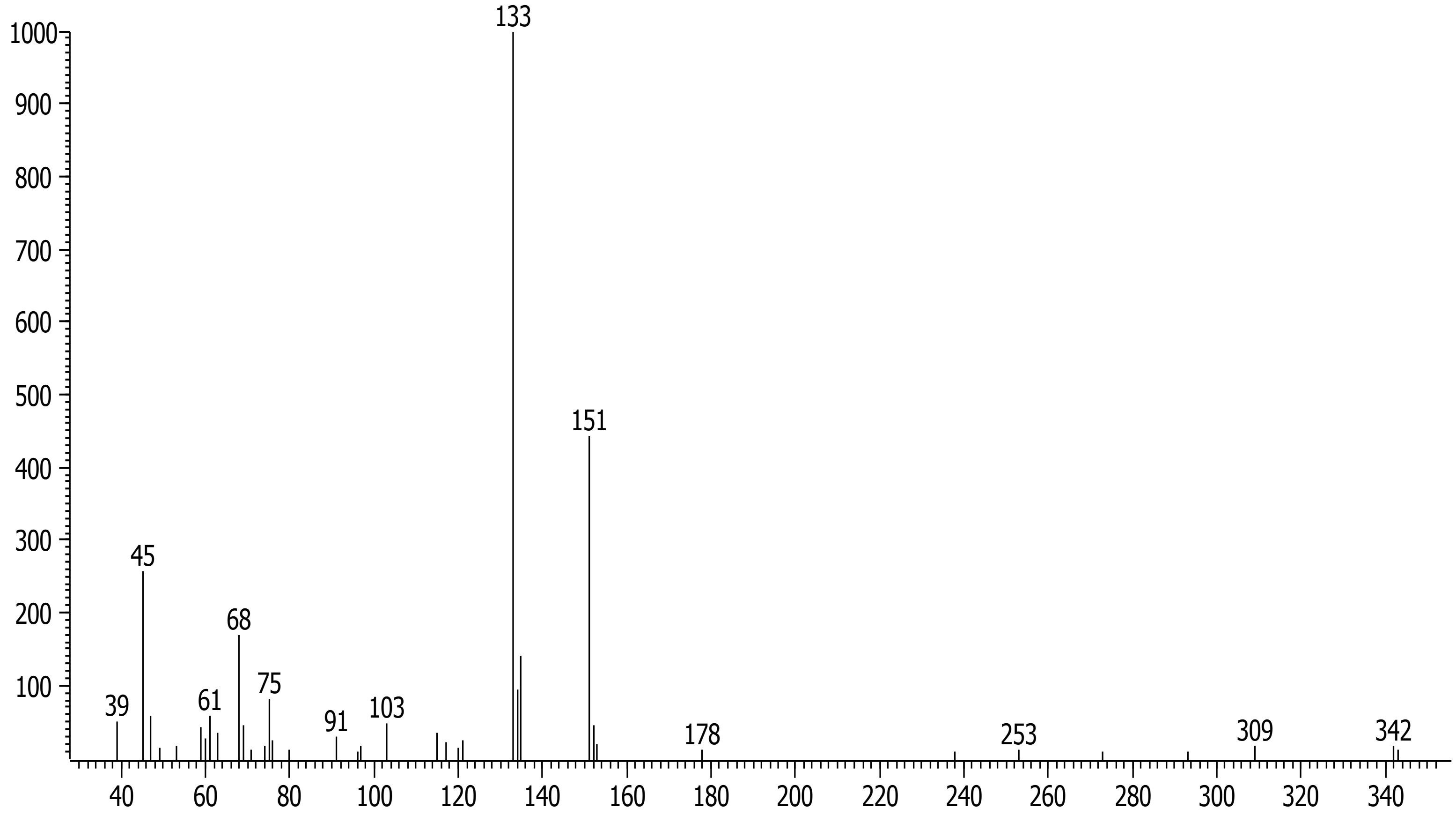

Octadecane, 6-methyl-

ANL258 Similarity: 85.9% RI: 1574

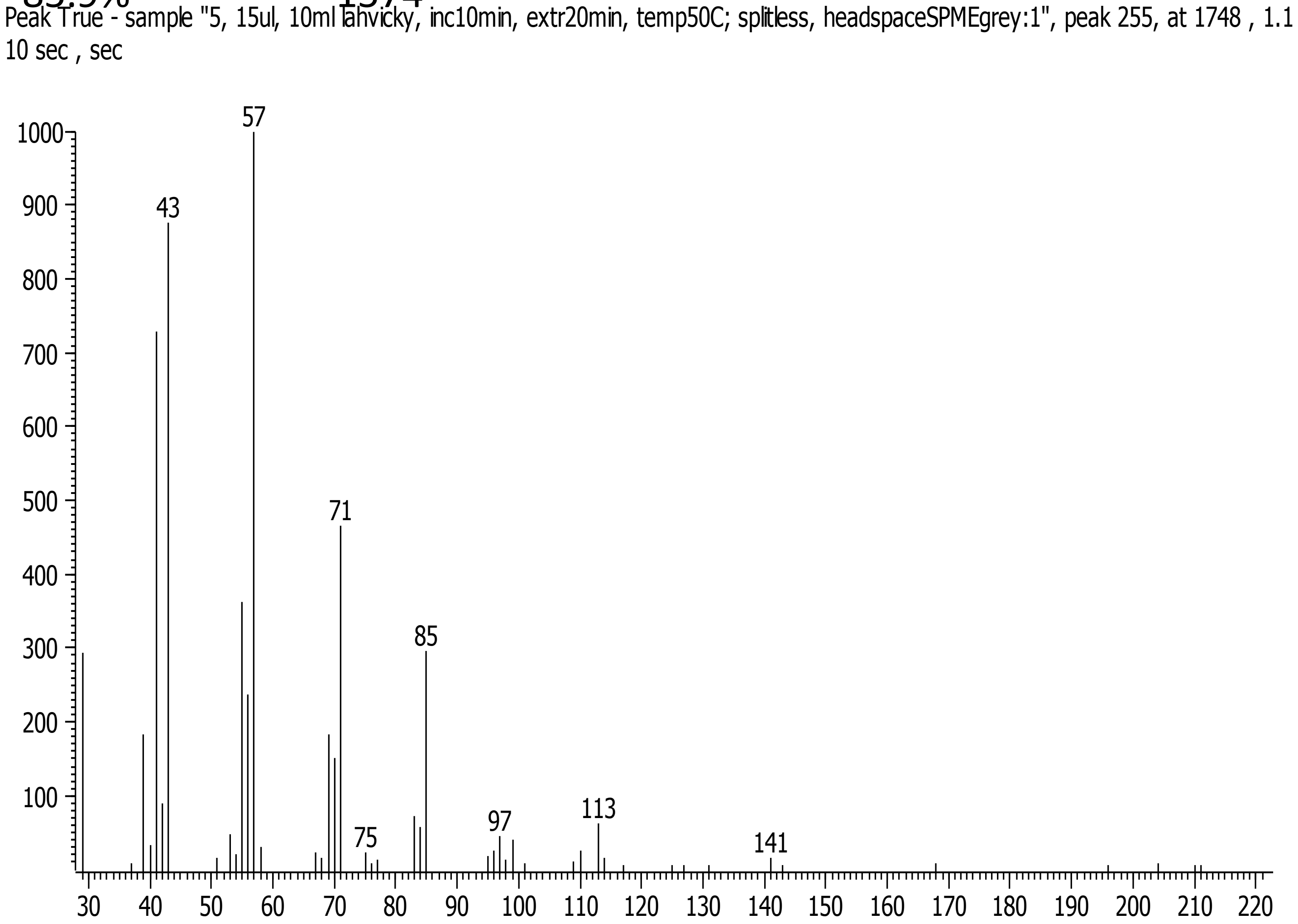

2-ACETYL-1-PYRROLINE

ANL101 Similarity: 88.3% RI: 926

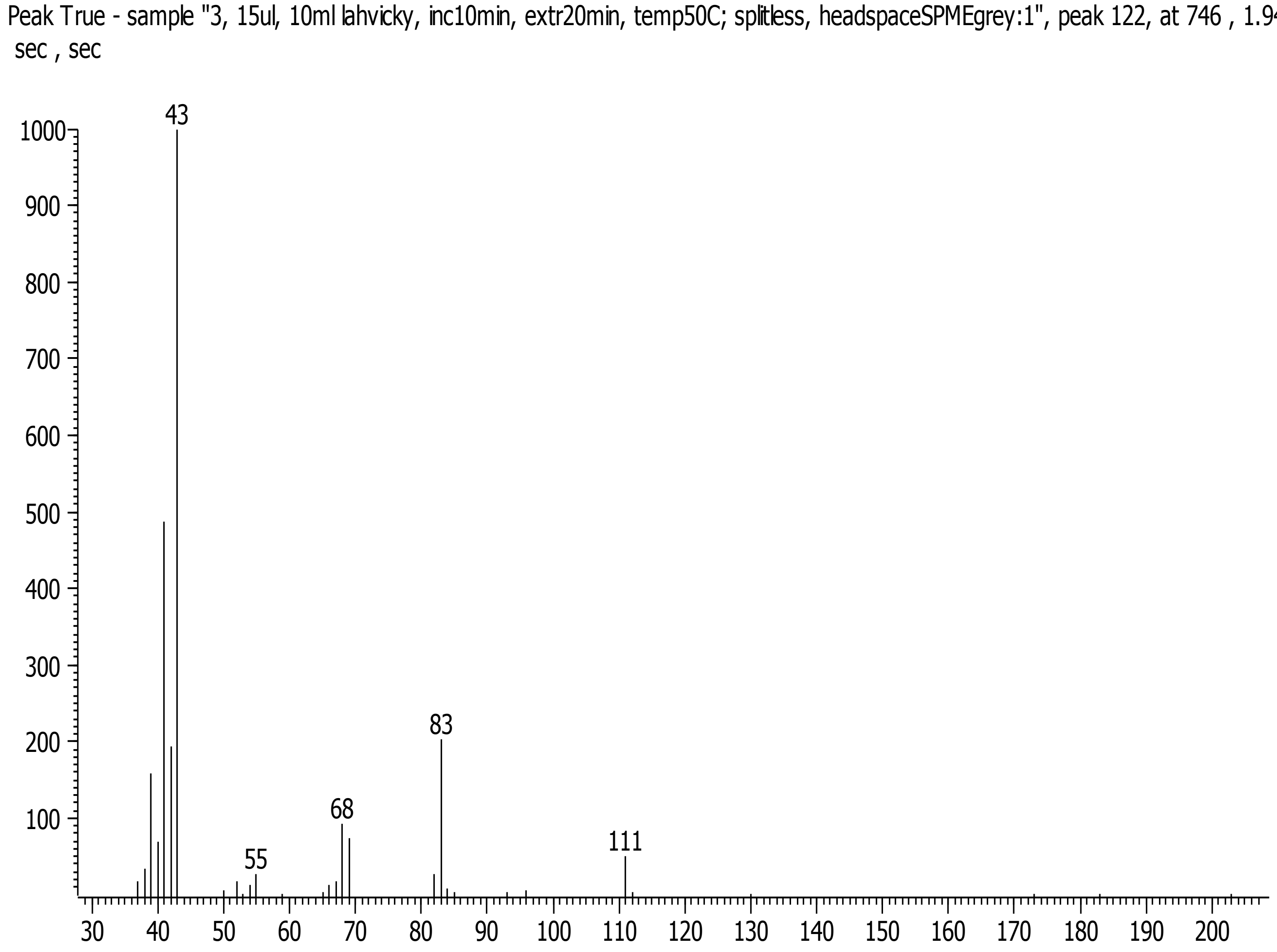

2-HEPTANONE

ANL94 Similarity: 91% RI: 893

Peak True - sample "8, 15ul, 10ml lahvicky, inc10min, extr20min, temp50C; splitless, headspaceSPMEgrey:1", peak 132, at 683 , 1.590 sec , sec

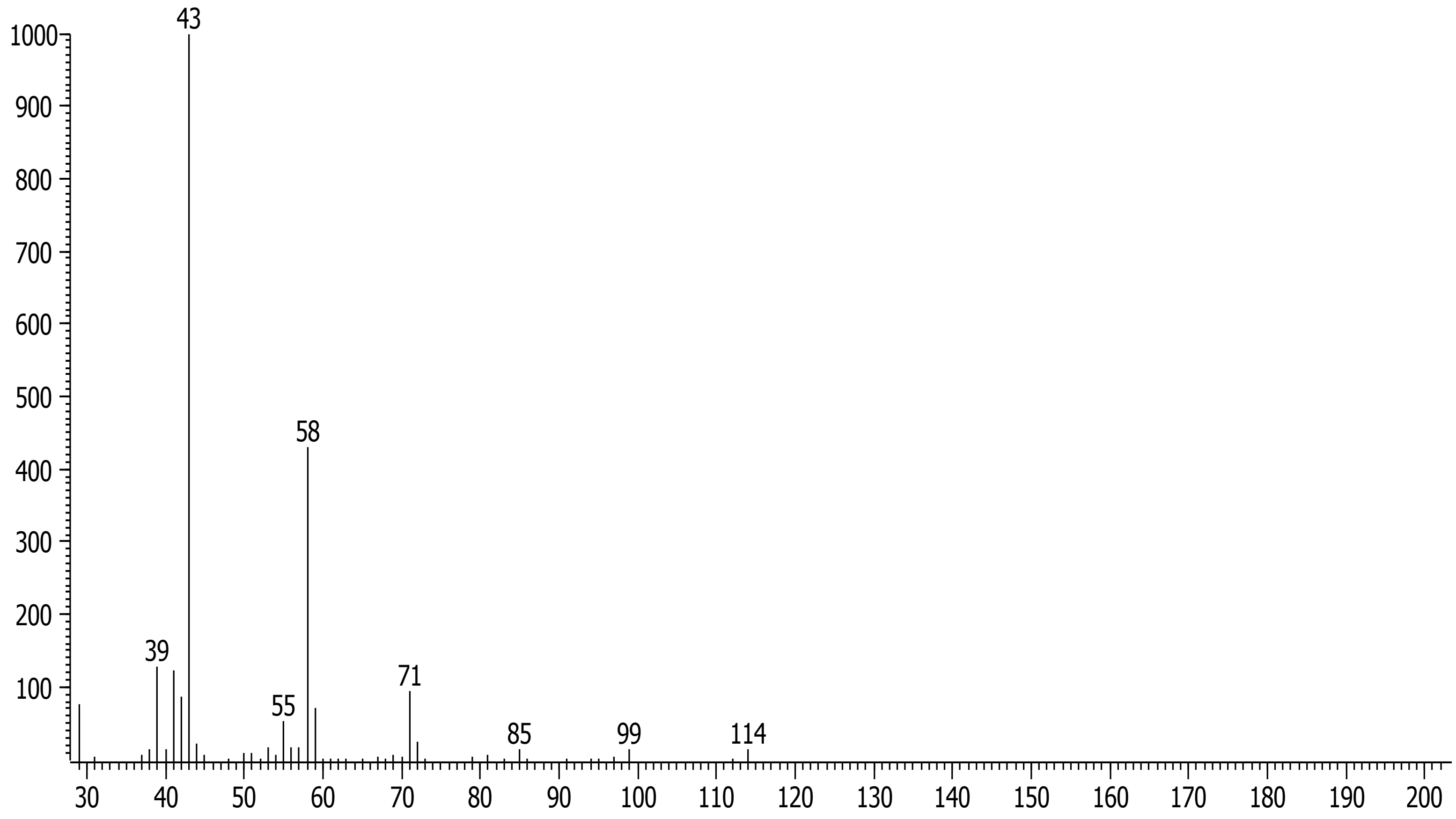

Analyte 293

ANL293 Similarity: % RI: 1796

Peak True - sample "9, 15ul, 10ml lahvicky, inc10min, extr20min, temp50C; splitless, headspaceSPMEgrey:1", peak 271, at 1901 , 1.490 sec , sec

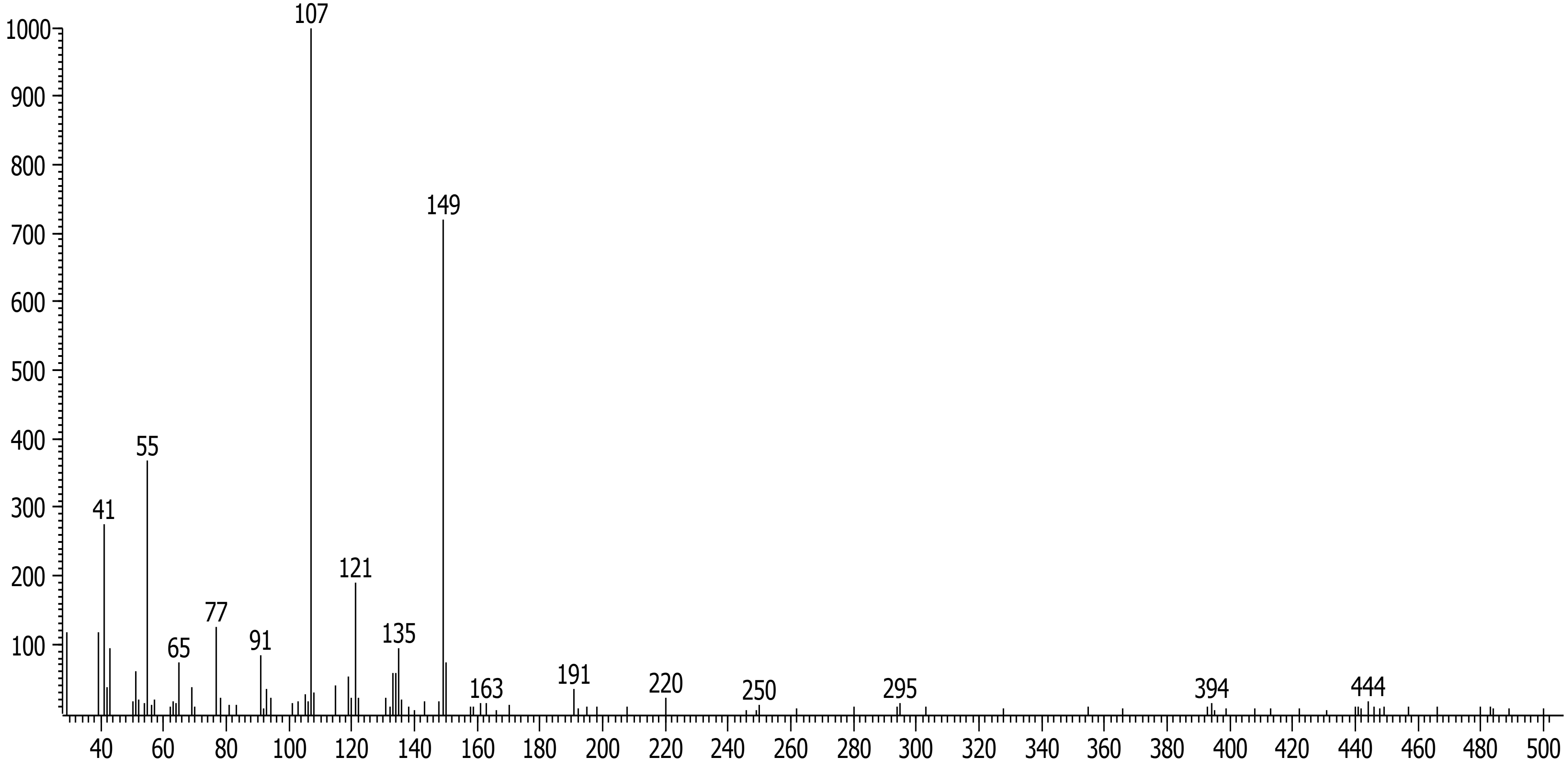

Analyte 218

ANL218 Similarity: % RI: 1354

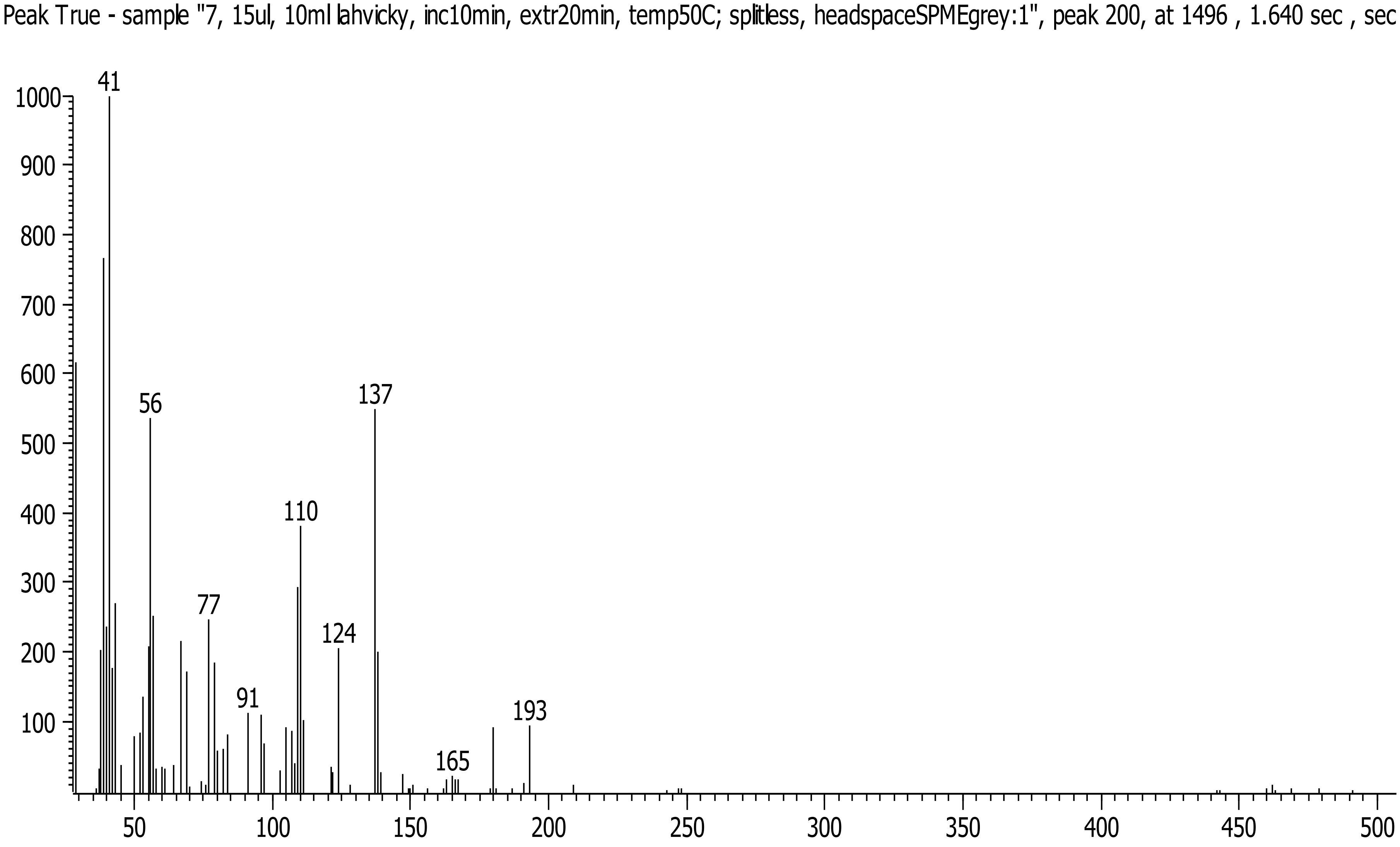

HEXANAL; 1-HEXANAL

ANL82 Similarity: 88% RI: 804

Peak True - sample "4, 15ul, 10ml lahvicky, inc10min, extr20min, temp50C; splitless, headspaceSPMEgrey:1", peak 106, at 527 , 1.540 sec , sec

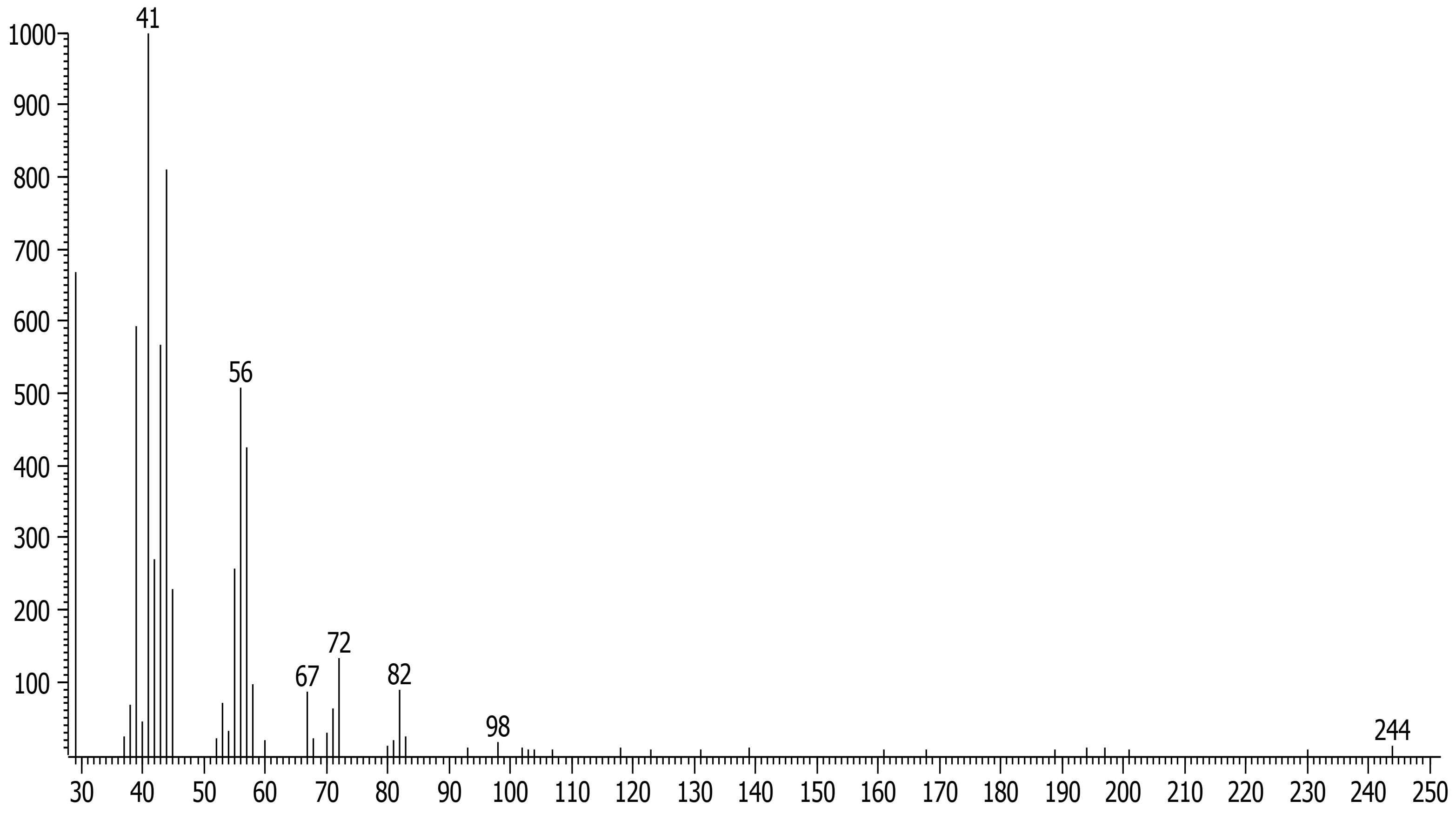

2-Pentenal, 2,4,4-trimethyl-

ANL89 Similarity: 73.8% RI: 838

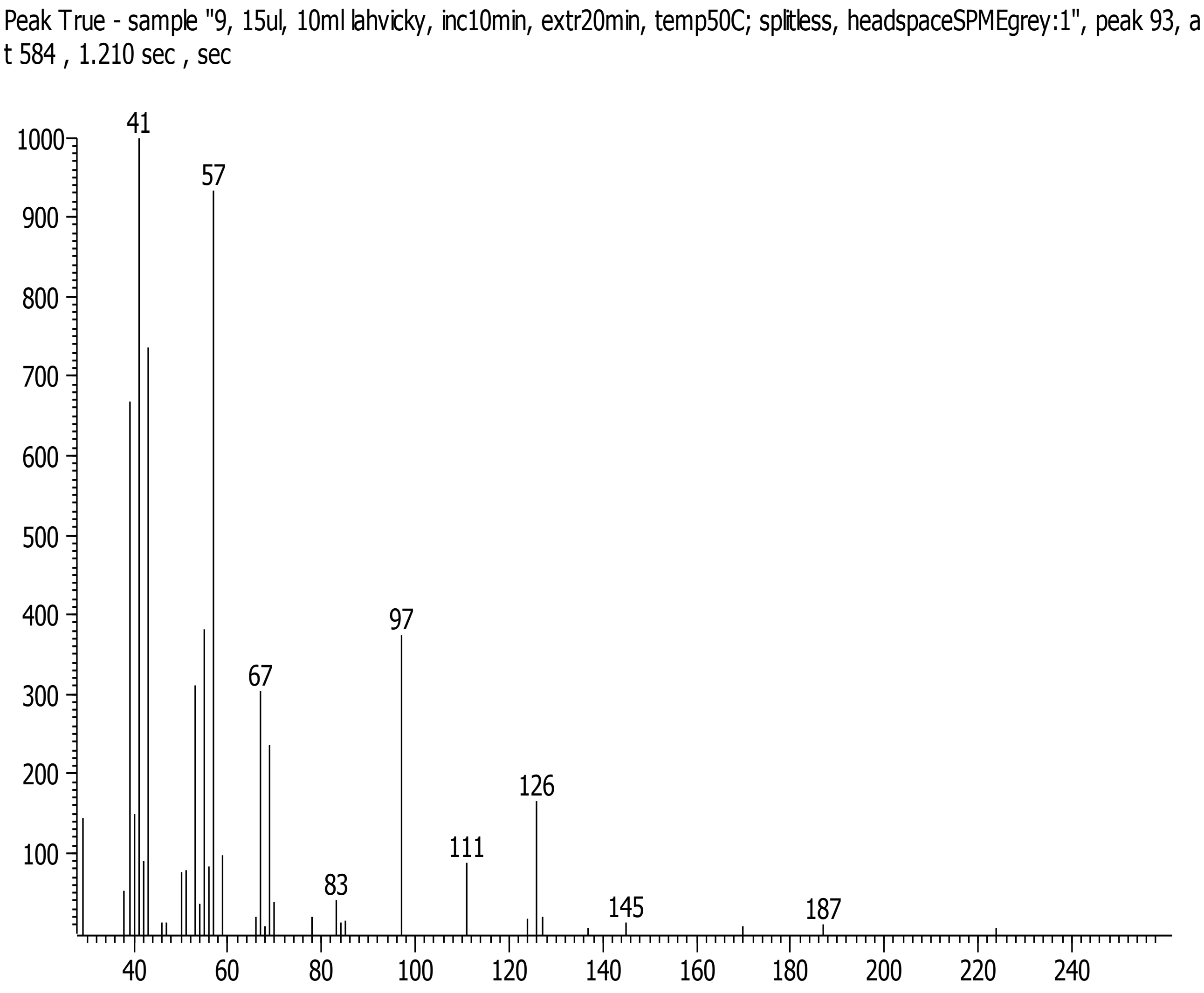

Tetracosane

ANL290 Similarity: 79% RI: 1758

Peak True - sample "9, 15ul, 10ml lahvicky, inc10min, extr20min, temp50C; splitless, headspaceSPMEgrey:1", peak 268, at 1895 , 1.090 sec , sec

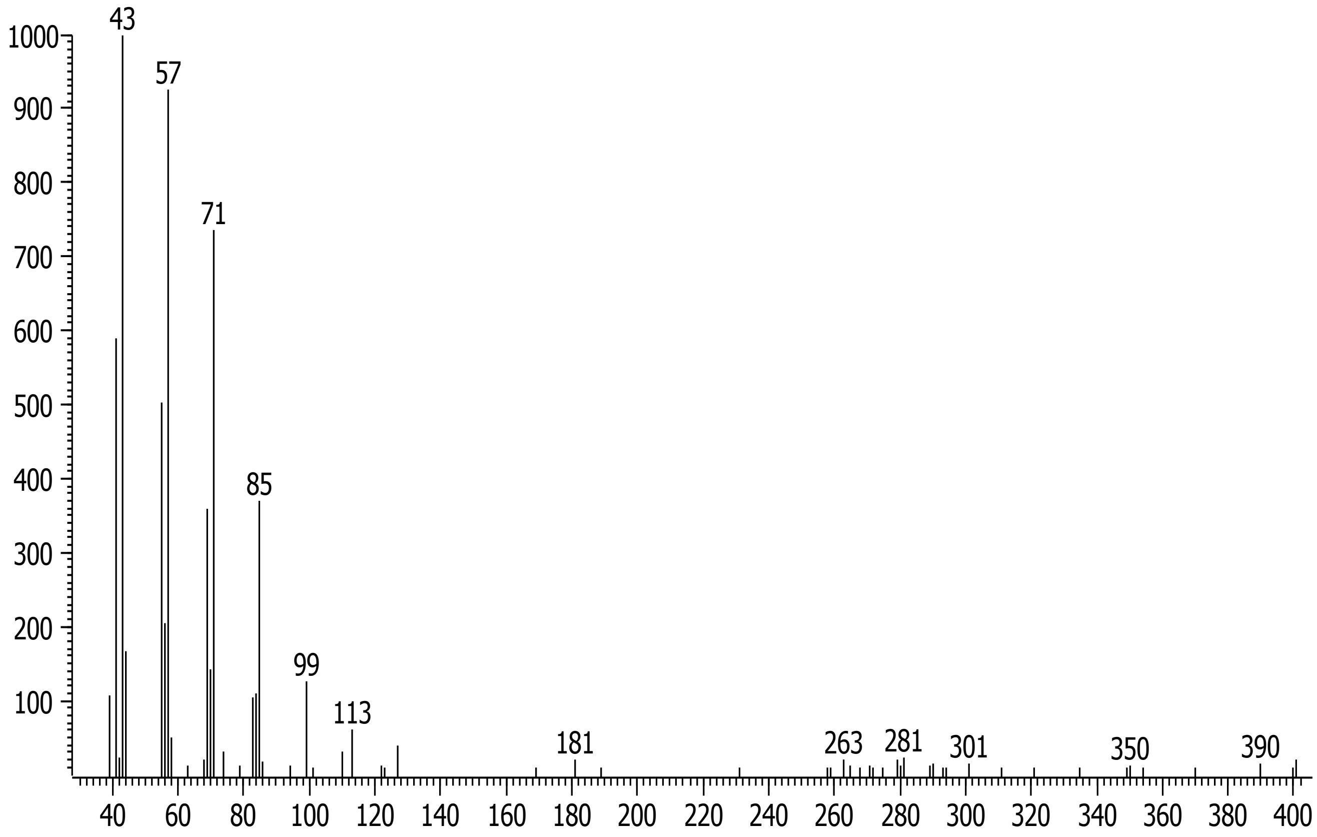

Oxime-, methoxy-phenyl-_

ANL117 Similarity: 35% RI: 979

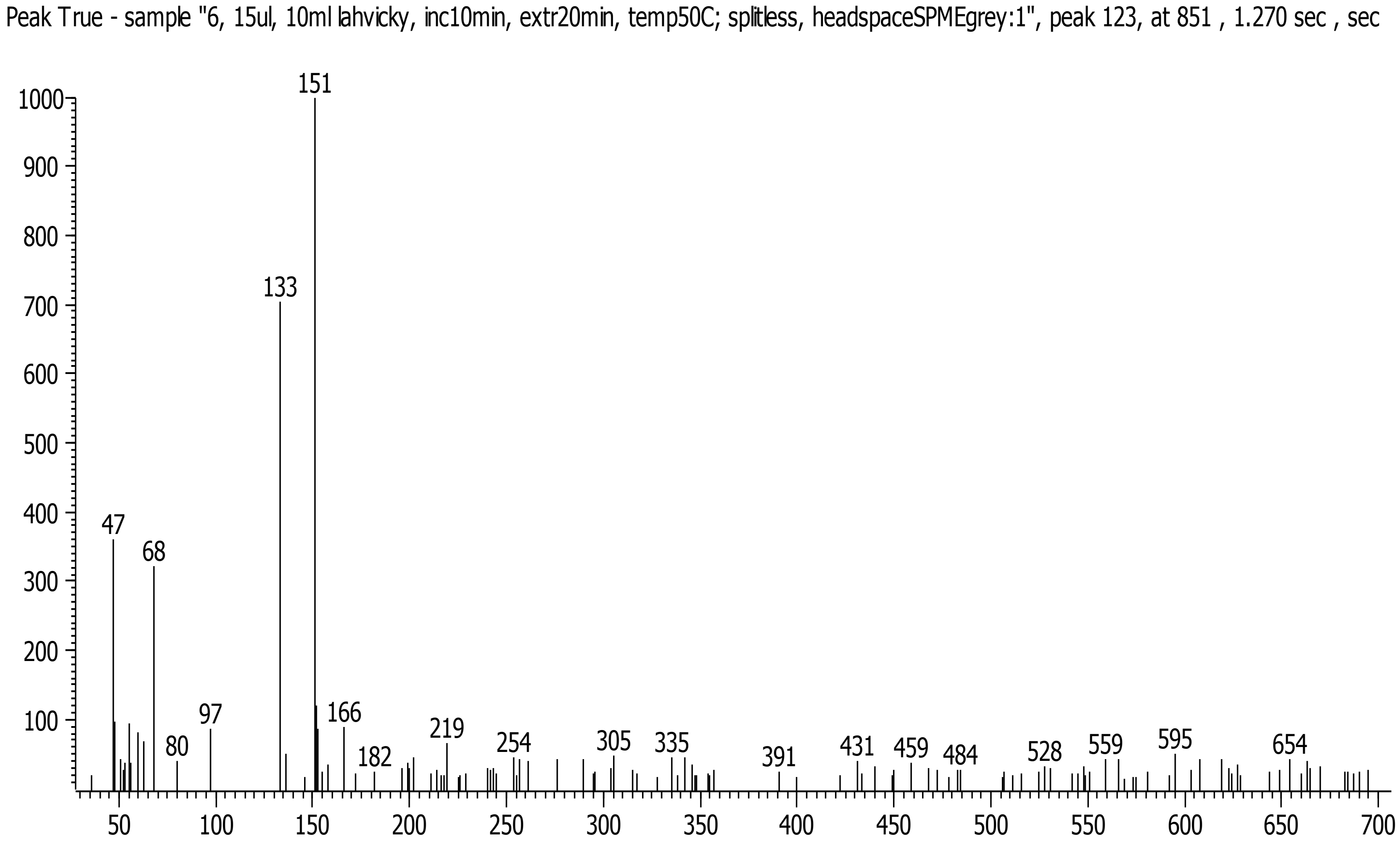

Analyte 301

ANL 301 Similarity: % RI: 1836

Peak True - sample "9, 15ul, 10ml lahvicky, inc10min, extr20min, temp50C; splitless, headspaceSPMEgrey:1", peak 278, at 1949 , 1.090 sec , sec

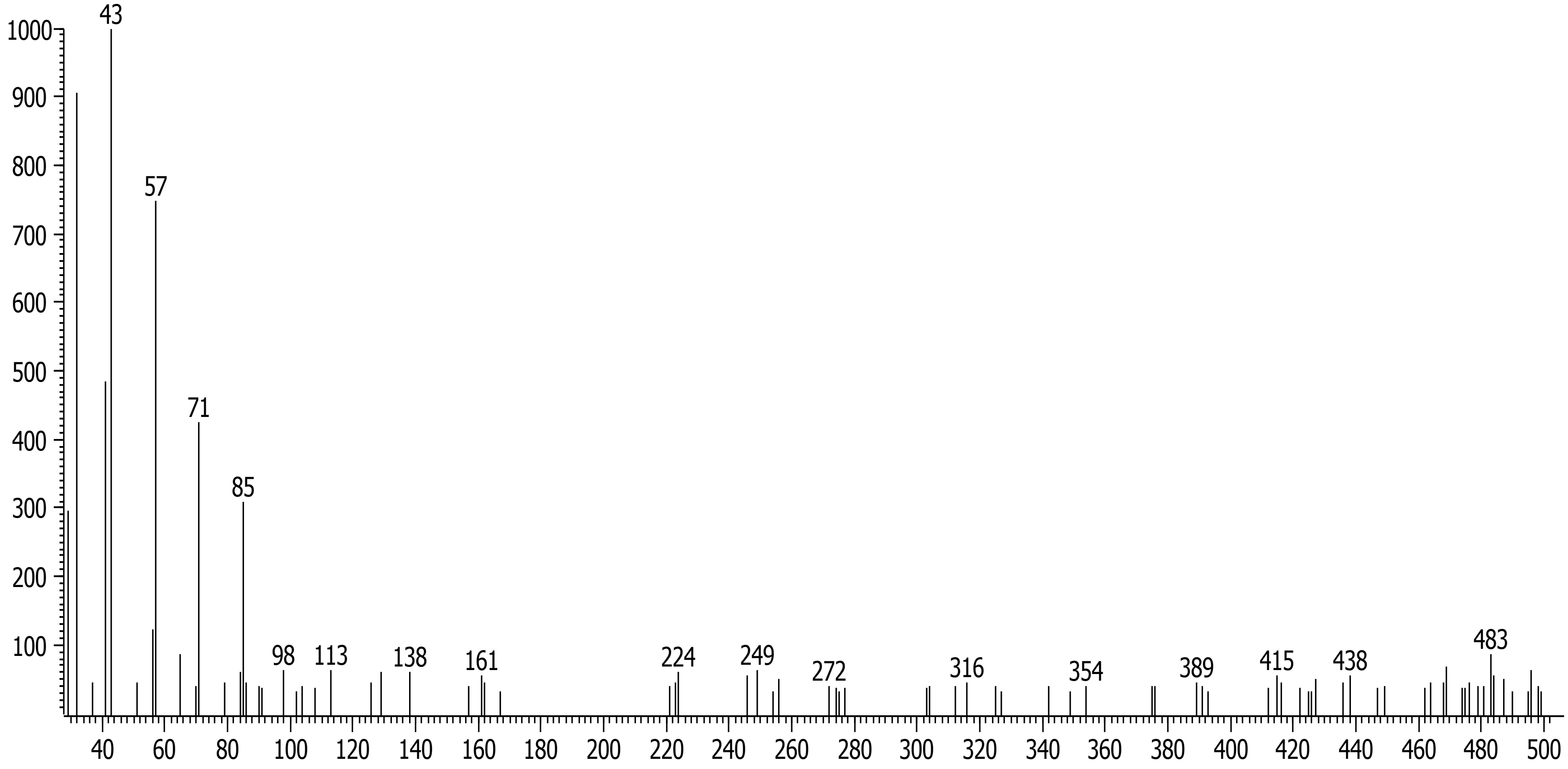

4-PENTENAL, 2-METHYL-

ANL260 Similarity: 64% RI: 1592

Peak True - sample "6, 15ul, 10ml lahvicky, inc10min, extr20min, temp50C; splitless, headspaceSPMEgrey:1", peak 242, at 1766 , 1.300 sec , sec

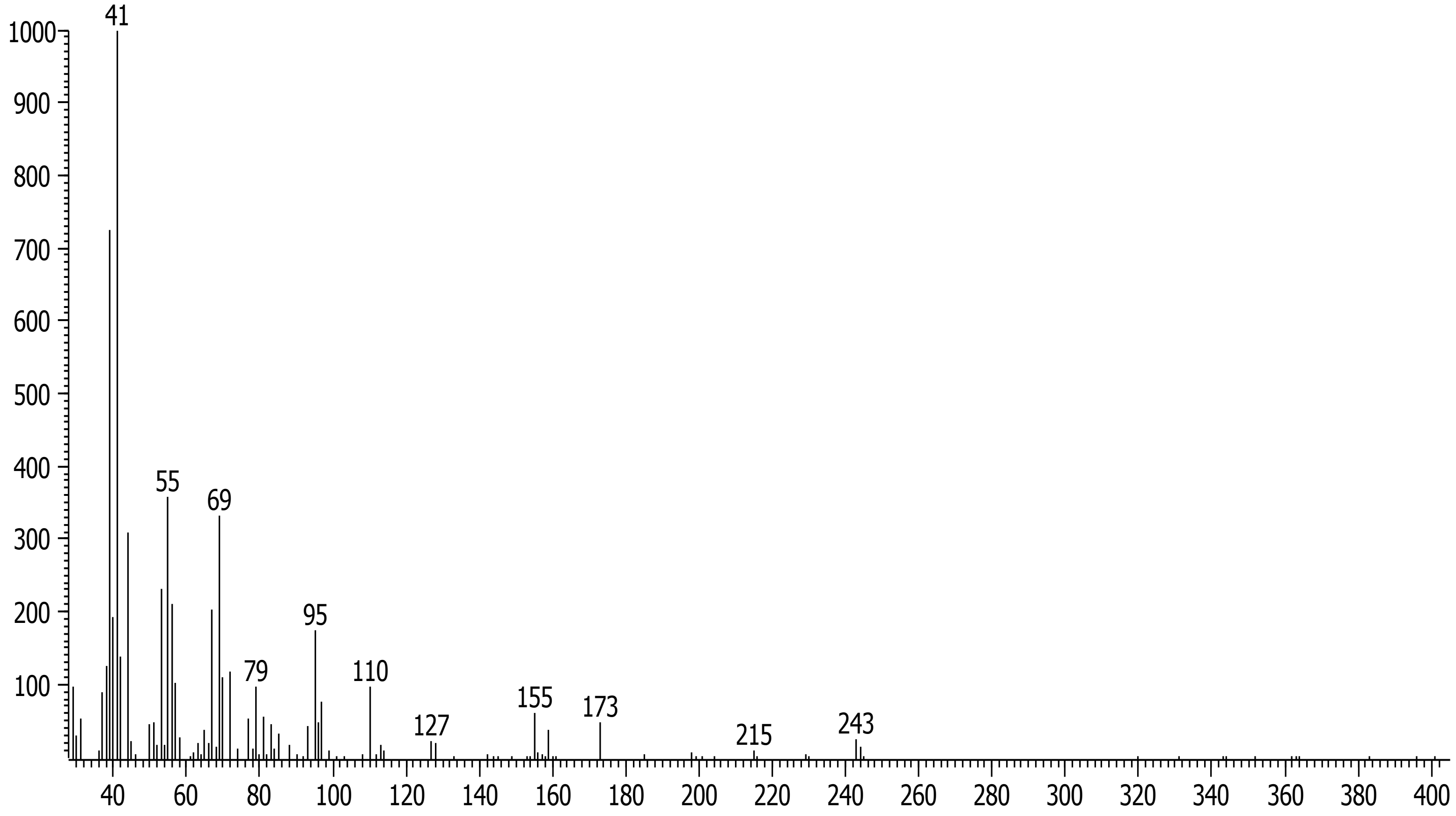

Acetophenone

ANL149 Similarity: 93% RI: 1076

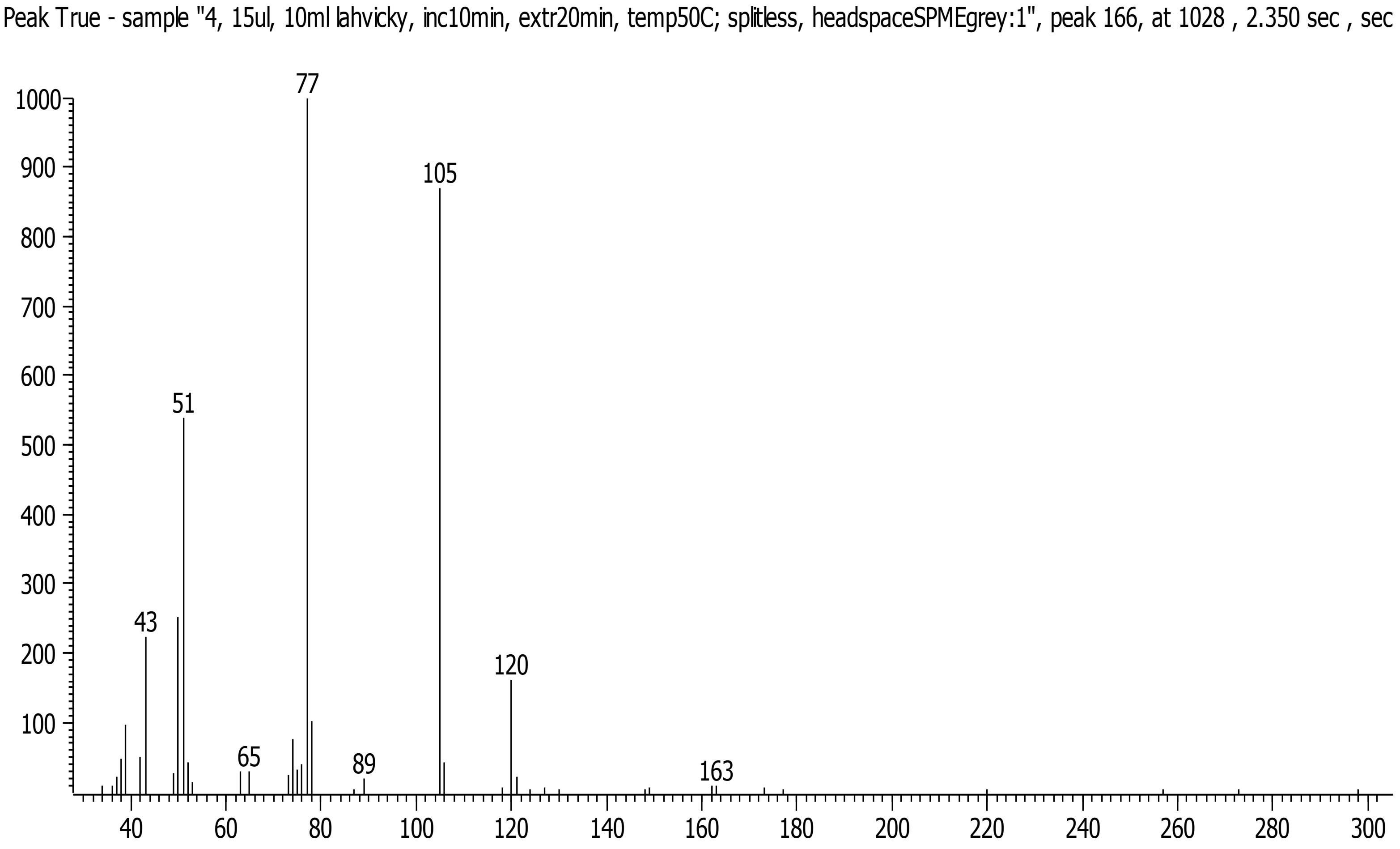

Supplement: Supplemental file S2 — Mass spectra of selected analytes. [file spectrum.02037-23-s0002.pdf]
